# Genomewide association analyses of fitness traits in captive‐reared Chinook salmon: Applications in evaluating conservation strategies

**DOI:** 10.1111/eva.12599

**Published:** 2018-03-05

**Authors:** Charles D. Waters, Jeffrey J. Hard, Marine S. O. Brieuc, David E. Fast, Kenneth I. Warheit, Curtis M. Knudsen, William J. Bosch, Kerry A. Naish

**Affiliations:** ^1^ School of Aquatic and Fishery Sciences University of Washington Seattle WA USA; ^2^ Conservation Biology Division Northwest Fisheries Science Center National Oceanic and Atmospheric Administration Seattle WA USA; ^3^ Department of Biosciences Centre for Ecological and Evolutionary Synthesis (CEES) University of Oslo Oslo Norway; ^4^ Yakama Nation Fisheries Toppenish WA USA; ^5^ Washington Department of Fish and Wildlife Olympia WA USA; ^6^ Oncorh Consulting Olympia WA USA

**Keywords:** captive rearing, conservation, domestication selection, genomewide association analysis, managed gene flow, random forest

## Abstract

A novel application of genomewide association analyses is to use trait‐associated loci to monitor the effects of conservation strategies on potentially adaptive genetic variation. Comparisons of fitness between captive‐ and wild‐origin individuals, for example, do not reveal how captive rearing affects genetic variation underlying fitness traits or which traits are most susceptible to domestication selection. Here, we used data collected across four generations to identify loci associated with six traits in adult Chinook salmon (*Oncorhynchus tshawytscha*) and then determined how two alternative management approaches for captive rearing affected variation at these loci. Loci associated with date of return to freshwater spawning grounds (return timing), length and weight at return, age at maturity, spawn timing, and daily growth coefficient were identified using 9108 restriction site‐associated markers and random forest, an approach suitable for polygenic traits. Mapping of trait‐associated loci, gene annotations, and integration of results across multiple studies revealed candidate regions involved in several fitness‐related traits. Genotypes at trait‐associated loci were then compared between two hatchery populations that were derived from the same source but are now managed as separate lines, one integrated with and one segregated from the wild population. While no broad‐scale change was detected across four generations, there were numerous regions where trait‐associated loci overlapped with signatures of adaptive divergence previously identified in the two lines. Many regions, primarily with loci linked to return and spawn timing, were either unique to or more divergent in the segregated line, suggesting that these traits may be responding to domestication selection. This study is one of the first to utilize genomic approaches to demonstrate the effectiveness of a conservation strategy, managed gene flow, on trait‐associated—and potentially adaptive—loci. The results will promote the development of trait‐specific tools to better monitor genetic change in captive and wild populations.

## 1 INTRODUCTION

There is considerable interest in applying the results of genomewide association analyses to conservation and management (Bernatchez, 2016; Bernatchez et al., 2017; Funk, McKay, Hohenlohe, & Allendorf, 2012; Garner et al., 2016; Harrisson, Pavlova, Telonis‐Scott, & Sunnucks, 2014; Hoffmann et al., 2015; Pearse, 2016; Shafer et al., 2015). In fact, identifying the genetic basis of fitness traits has already provided key information for these purposes, including an improved understanding of adaptive divergence (Brieuc, Ono, Drinan, & Naish, 2015; Hornoy, Pavy, Gerardi, Beaulieu, & Bousquet, 2015), the discrimination of ecotypes within a panmictic species (Pavey et al., 2015), detecting polygenic selection to aquatic pollutants (Laporte et al., 2016), and the development of a marker panel for routine trait and population monitoring (Aykanat, Lindqvist, Pritchard, & Primmer, 2016; Barson et al., 2015). As the availability of genomic resources improves, it is important to explore how markers identified by association analyses on natural populations might be applied in different contexts.

Trait‐associated markers have significant potential to inform the management of captive breeding programs. Captive breeding remains one of the primary options for the conservation of threatened populations and species (e.g., Conde, Flesness, Colchero, Jones, & Scheuerlein, 2011; Griffiths & Pavajeau, 2008; Horne, Hervert, Woodruff, & Mills, 2016; Landa et al., 2017). However, this approach is also controversial, because associated genetic and phenotypic changes may decrease the fitness of captive individuals when they are released into the wild and, consequently, reduce restoration success (Christie, Ford, & Blouin, 2014; Frankham, 2008; Jule, Leaver, & Lea, 2008). Surveys of variation at trait‐associated loci would improve our understanding of how captive breeding affects potentially adaptive genetic variation and would help to identify traits, and even specific alleles, that may drive observed fitness reductions (e.g., Bateson et al., 2016). Trait‐based monitoring could also inform policy decisions that aim to minimize negative effects of captive breeding, including practices to reduce domestication selection.

Population supplementation using hatcheries, a form of captive breeding, is part of many recovery plans for Pacific salmon on the West Coast of North America, where numerous populations have declined or been extirpated in the last century (National Research Council 1996). In these programs, adult salmon are brought into the hatchery for reproduction, and their offspring are reared in captivity for up to 2 years before they are released into the wild as seaward migrants. Despite spending only a portion of their lives in captivity, hatchery‐reared salmon have exhibited significant differences from their wild counterparts, including reduced reproductive success (Christie et al., 2014), differences in growth rate and morphology (Busack, Knudsen, Hart, & Huffman, 2007; McGinnity et al., 2003), and increased vulnerability to predation (Fritts, Scott, & Pearsons, 2007). One practice that has been widely adopted to mitigate genetic and phenotypic risks of hatchery rearing is the integration of wild or natural‐origin (born in the wild but may have hatchery ancestry) fish into hatchery broodstock (Mobrand et al., 2005; Paquet et al., 2011). This “integrated” approach, which we refer to as managed gene flow, contrasts with the traditional “segregated” strategy where only hatchery‐origin fish are used as broodstock. Theoretical studies based on genetic models predict that managed gene flow reduces, but does not eliminate, the effects of genetic drift, inbreeding, and domestication selection that may occur due to captive breeding (Baskett & Waples, 2013; Duchesne & Bernatchez, 2002; Ford, 2002; Lynch & O'Hely, 2001). In addition, we recently provided the first empirical genetic evidence demonstrating the benefits of managed gene flow in captive‐reared fish. Specifically, genomewide divergence over four generations of captive rearing was reduced in an integrated hatchery population of Chinook salmon when compared to a program based on broodstock segregation (Waters et al., 2015, 2017), signifying that the overall genetic risks of captive rearing may be reduced through gene flow. Here, we extend this work by using loci associated with several fitness‐related traits to explore how the integrated and segregated management strategies affect genetic variation at trait‐associated, and potentially adaptive, loci.

**Figure 1:**
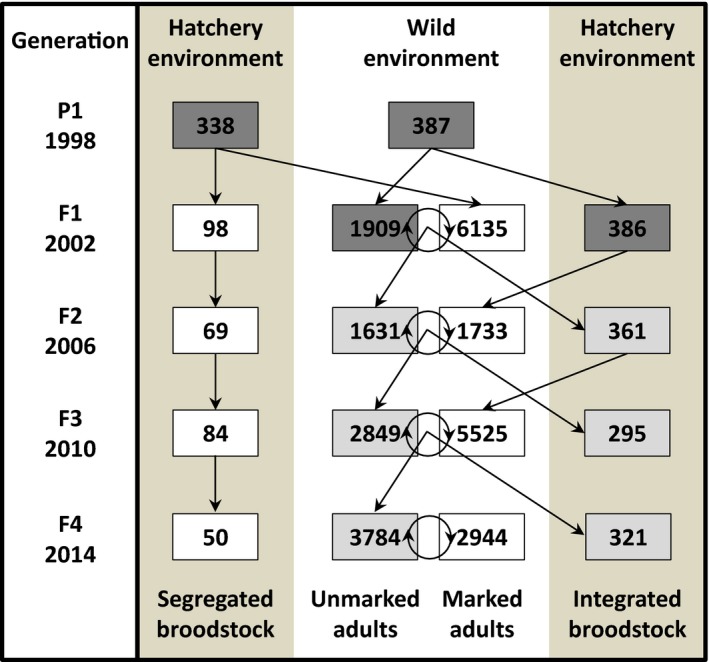
Schematic illustrating the initiation (the founding *P*_1_ generation) and subsequent propagation (*F*_1_–*F*_4_ generations) of the integrated and segregated hatchery lines of anadromous Chinook salmon at the Cle Elum Supplementation and Research Facility (modified from Waters et al., 2015). Each box denotes the number of spawners (wild environment) and the number of broodstock (hatchery environment) for each year surveyed. Linear arrows indicate the contribution of wild spawners or hatchery broodstock to the subsequent generation. Circular arrows represent unobserved mating between wild‐born (unmarked) and hatchery‐born (marked) spawners in the wild environment. Fish from the two lines are differentially marked, so only hatchery‐born fish from the integrated line are permitted to spawn in the wild. Dark gray boxes represent wild adults, light gray boxes represent natural‐origin adults with hatchery, wild, or hybrid ancestry, and white boxes represent adults born in the hatchery

The spring‐run Chinook salmon hatchery program at the Cle Elum Supplementation and Research Facility (CESRF) in Cle Elum, Washington, USA, is unique in that it maintains an integrated hatchery line and a segregated hatchery line, both of which were derived from the same wild population at the same time (Figure 1). Importantly, tissue samples for DNA and phenotypic data have been collected from every adult fish used as broodstock since the inception of the program in 1997. Many of the phenotypic traits measured—length, weight, and dates of return to freshwater and maturation—are correlated with individual fitness (e.g., Kodama, Hard, & Naish, 2012; Schroder et al., 2010; Thorpe, Miles, & Keay, 1984). In addition, these traits have significant additive genetic variation on which selection can act (Carlson & Seamons, 2008; Hard, 2004) and can differ between hatchery and wild populations (e.g., Ford et al., 2006; Hoffnagle, Carmichael, Frenyea, & Keniry, 2008; Knudsen et al., 2006). Therefore, studying the genetic basis of these specific traits may provide a better understanding of how domestication selection acts and, in turn, reveal possible mechanisms underlying the reduced fitness of hatchery‐origin fish after they are released into the wild.

Here, we used individual‐based data from the two hatchery lines at CESRF to (i) identify loci associated with six fitness‐related traits that have been measured in returning adults across four generations and (ii) determine if managed gene flow successfully limited divergence at trait‐associated loci relative to a segregated management approach. Specifically, we first characterized the genomic basis of date of return to freshwater spawning grounds (return timing), fork length and weight at return, age at maturity, spawn timing, and daily growth coefficient using 9108 restriction site‐associated (RAD) loci and a genomewide association analysis suitable for polygenic traits, random forest (Breiman, 2001). Loci associated with each trait were then annotated to provide biological context for the genotype–phenotype associations. Next, genetic variation at loci associated with each trait was compared between each generation of the integrated and segregated hatchery lines to determine if managed gene flow limited divergence. We also compared the genomic positions of trait‐associated loci and highly diverged loci—interpreted as signatures of adaptive divergence—that had been previously identified in the two lines (Waters et al., 2015, 2017). Overlap between the two groups of loci was used to infer which traits may have responded to domestication selection. This study represents one of the first efforts to evaluate the effectiveness of a conservation strategy by examining loci linked to multiple traits within a nonmodel organism. The results provide molecular tools for monitoring captive populations and could inform management practices to reduce possible adverse effects of captive rearing.

## 2 MATERIALS AND METHODS

### 2.1 Study system

The ecological background of the study population and the initiation of the integrated and segregated hatchery lines at CESRF have been described in previous publications (Fast et al., 2015; Knudsen et al., 2006; Waters et al., 2015). Briefly, wild adults returning from the ocean to their freshwater spawning grounds were collected for founding broodstock (Figure 1) from the upper Yakima River, WA, USA, population from 1997 to 2002 as they passed the Roza Dam Adult Monitoring Facility (RAMF), located 90 river kilometers south of CESRF. Broodstock were collected at random over the entire duration of the salmon run and transported to CESRF, where they were held in concrete raceways until maturation. Mature adults were spawned at random following a 3 × 3 factorial mating design (when possible). Fertilized eggs, fry, and juveniles were reared at CESRF for approximately 16 months, after which they were transferred to three acclimation sites; these sites were designed to expand the spatial influence of supplementation efforts while also enabling related research. Fish were acclimated for 2 months, at which point they were allowed to volitionally begin their migration to the ocean. A majority (≥75%; Knudsen et al., 2006) of Chinook from this population spend 2 years in the ocean and return at age four to reproduce.

In 2002, both wild and first‐generation hatchery adults were spawned to create the integrated (INT) and segregated (SEG) hatchery lines, respectively (Figure 1). The integrated line uses only fish born in the wild as broodstock, and all returning adults from this line are allowed to spawn in the river. In contrast, only returning hatchery‐origin fish are used as broodstock in the segregated line, and SEG adults are not allowed to reproduce naturally; fish from the two lines are differentially marked for external identification, so all SEG adults are removed from the system at the RAMF. Therefore, the integrated line receives one generation of exposure to hatchery conditions while the segregated line is exposed every generation (Figure 1). Broodstock collection, spawning, and rearing procedures for both lines are conducted in the same manner as the founding generation.

### 2.2 Tissue sample and phenotypic data collection

Tissue samples for DNA were collected from all fish during spawning at CESRF and stored in 100% ethanol. Five generations were subsampled for this study: the 1998 wild founders (second founding year; *P*_1_ Founders) and hatchery brood years 2002 (*F*_1_ Wild and *F*_1_ Hatchery), 2006 (*F*_2_ INT and *F*_2_ SEG), 2010 (*F*_3_ INT and *F*_3_ SEG), and 2014 (*F*_4_ INT and *F*_4_ SEG).

Phenotypic traits of all returning adults were measured annually upon arrival at RAMF and, for those collected as broodstock, during spawning at CESRF. Six traits were analyzed in this study (Table 1). Ages at maturity of hatchery‐origin broodstock were determined from passive integrated transponder or coded‐wire tags, which denote the brood year of each fish, while those of natural‐origin broodstock and hatchery‐origin fish without tags were determined from growth rings on their scales (Clutter & Whitesel, 1956). Ages of fish not used as broodstock (i.e., those allowed to spawn naturally or removed from the system) were estimated with relatively high accuracy (up to 90%; C. Knudsen, pers. comm.) from body size measurements at RAMF. Return timing corresponds to day of arrival at RAMF. Spawn, or maturation, timing of all broodstock was estimated weekly at CESRF by manually checking for gonadal ripeness. Fork length and weight were measured at both RAMF and upon spawning at CESRF. However, only measurements from RAMF were analyzed here to minimize possible influences of the kype, a secondary sex trait in males that develops during maturation and may increase jaw length up to 50% (Fleming, 1996), and of time spent in the hatchery after collection at RAMF. Daily growth coefficient (DGC) was calculated according to Cho (1992) and Dupont‐Nivet et al. (2010):

(1) $$\text{DGC}=100\times \left[\left({\left(\text{final weight}\right)}^{1/3}-{\left(\text{initial weight}\right)}^{1/3}\right)/\text{days}\right]$$

where initial weight was the weight at RAMF, final weight was weight at spawning, and days was the number of days between arrival at RAMF and spawning at CESRF. DGC has been shown to be more independent of initial weight than other measures, such as specific growth rate, and is thus better for growth rate comparisons (Cho, 1992). Here, DGC provided a measure related to weight lost between RAMF and CESRF and was used to infer if the two hatchery lines responded differently to holding conditions in the tanks at CESRF (e.g., if fish from the segregated line exhibited lower daily growth coefficients and levels of stress due to holding); such differences may indicate adaptation to captivity.

**Table 1:** Individual traits measured in adult Chinook salmon returning to the upper Yakima River. Fish are measured at the Roza Dam Adult Monitoring Facility (RAMF) and again if used as broodstock at the Cle Elum Supplementation and Research Facility (CESRF). Measurements from RAMF were used in the analyses for fork length and weight

Trait category	Traits (units)	Locations measured	
Life history	Age at maturity (years)	RAMF	CESRF
	Return timing (day of year)	RAMF	
	Spawn timing (day of year)		CESRF
Morphometric	Fork length (cm)	RAMF	CESRF
	Weight (kg)	RAMF	CESRF
Growth	Daily growth coefficient (no units)	RAMF to CESRF	

### 2.3 DNA sequencing and genotyping

DNA from tissue samples was extracted using DNeasy Blood & Tissue kits (Qiagen, Valencia, CA, USA) following the animal tissue protocol. RAD libraries (Baird et al., 2008) were prepared using the restriction enzyme *Sbf1* and by pooling 24–36 bar‐coded individuals per lane (28 lanes total). Single‐read (100 bp) sequencing was performed using an Illumina HiSeq2000.

RAD sequences were processed using *Stacks* (v. 1.09; Catchen, Hohenlohe, Bassham, Amores, & Cresko, 2013). First, reads were demultiplexed and trimmed to 74 base pairs using *process_radtags*, as sequencing errors increased after this length. Reads were then aligned to a reference database of 48528 putatively nonduplicated RAD loci for Chinook salmon, 6350 of which are positioned on a linkage map (Brieuc, Waters, Seeb, & Naish, 2014), using *Bowtie* (v. 0.12.8; Langmead, Trapnell, Pop, & Salzberg, 2009) with the “best” option and allowing up to three mismatches. Loci for each individual were identified using *pstacks* in *Stacks* with the bounded‐error SNP calling model, default error rates, and a minimum stack depth of 10 reads. A catalog of loci was then constructed from the five most‐sequenced individuals per population using *cstacks*; a subset of individuals was used to reduce the risk of including false polymorphisms in the catalog (e.g., Hess, Zendt, Matala, & Narum, 2016; Narum et al., 2017; Nichols, Kozfkay, & Narum, 2016; Waters et al., 2015). Loci of individuals were matched to the catalog using *sstacks*, and genotypes were aggregated using *populations*.

After processing with *Stacks*, all biallelic loci were re‐genotyped with a custom Python script to minimize potential bias in maximum likelihood genotype calls due to differences in read depth between two alleles at a locus. The custom script (Brieuc et al., 2014) designated a genotype as heterozygous if both alleles had a minimum depth of two and a combined read depth >10. Loci were then screened and retained if they had a minor allele frequency ≥0.05 in at least one population. Individuals were removed if they had ≥50% missing genotypes across all loci. Then, individual loci were removed if they were not genotyped in at least 50% of the individuals in each population.

Missing genotypes in the final data set were subsequently imputed using *fastPhase* (Scheet & Stephens, 2006), because association analyses based on random forest cannot process missing data. Deviations from Hardy–Weinberg equilibrium were then identified using the exact test in *Genepop* (v. 4.1; Raymond & Rousset, 1995) with default parameter values. Loci that did not conform to expected Hardy–Weinberg proportions in two or more populations were removed (e.g., Benestan et al., 2015; Nichols et al., 2016), as deviations may arise from factors such as genotyping errors or the inclusion of paralogous sequence variants that remain after the whole genome duplication event in salmonids (Allendorf & Thorgaard, 1984). However, we acknowledge that this conservative approach may also remove loci of interest, such as those responding to domestication selection.

### 2.4 Inferring positions of unmapped loci

To increase the number of loci positioned on the Chinook linkage map, positions of unmapped loci were inferred by alignments to the rainbow trout (Berthelot et al., 2014) and Atlantic salmon (Lien et al., 2016) genomes, similar to the approach developed by Sutherland et al. (2016) and employed by Narum et al. (2017). First, all nonduplicated loci on the Chinook salmon linkage map (*n* = 6,350, Brieuc et al., 2014) and unmapped loci in the final data set were aligned to both genomes using *Bowtie 2* (v. 2.2.9; Langmead & Salzberg, 2012) with default parameters. Next, *Bowtie 2* output files were converted from SAM to BAM format using *SAMtools* (Li et al., 2009), and then from BAM to BED format using *bedtools* (v. 2.26.0; Quinlan & Hall, 2010). The *closest* function in *bedtools* then identified high‐quality alignments (mapping quality ≥10) of mapped and unmapped Chinook loci that were within 100 kb of each other. A distance of 100 kb was chosen because mapped loci with identical linkage map positions frequently aligned to the rainbow trout or Atlantic salmon genomes within 100–300 kb of each other. Unmapped loci were assigned the same linkage map positions as the closest mapped loci.

### 2.5 Random forest analyses

Loci associated with the phenotypic traits were identified by random forest, an approach suitable for simple and polygenic traits (Breiman, 2001). Unlike traditional approaches, random forest provides a nonparametric framework that can simultaneously incorporate many loci, thus enabling the identification of suites of loci that explain substantial phenotypic variation collectively but may not display significant associations individually.

Random forest, like other methods, can be confounded by population structure and additional factors (Stephan, Stegle, & Beyer, 2015; Zhao et al., 2012). Here, the approach of Zhao et al. (2012) was used to correct for the possible confounding factors of hatchery line, sex, age, year, and the coordinate of individuals on PC1 from a principal components analysis of the genotypes. Phenotypes and genotypes at all loci (one locus at a time) were regressed against these potential confounding factors using linear regression models in R (R Core Team 2016). The models explained any variation in the phenotypes and genotypes that may be due to differences between the two hatchery lines or any of the other factors. The residuals of the models, representing the “corrected” phenotypes and genotypes, were then analyzed by random forest to identify loci strictly associated with the traits of interest. When age at maturity was the response variable, genotypes were corrected for all possible confounding factors (except age), but phenotypes were not corrected to maintain their discrete (categorical) distribution, rather than transform them into continuous variables.

Random forest analyses were conducted using the R package *randomForest* (Liaw & Wiener, 2002). The *P*_1_ founders were excluded so that each year analyzed comprised only samples from the integrated and segregated hatchery lines (i.e., paired design). Age, as a discrete trait with an unequal distribution across values, was analyzed using balanced classification trees (specified with the *strata* and *sampsize* parameters in the *randomForest* function). Regression trees with default parameter settings were employed for the five continuous traits. For all traits, three independent forests were first grown using all loci. The optimal number of trees (*ntree* parameter) per forest was determined when the correlation of locus importance values between forests was relatively high (>0.8). Initial importance values for loci were then estimated by averaging values from the three forests. Next, three forests were grown on subsets of the most important loci (e.g., top 1%, top 2%, etc.) to determine a candidate group of loci that explained the highest proportion of phenotypic variation observed (for the five continuous traits) or had the lowest out‐of‐bag classification error rate (for age at maturity). The backward purging approach (Holliday, Wang, & Aitken, 2012) was then conducted on an expanded group of candidate loci (i.e., used top 1.5% of loci if the top 1% of loci appeared to explain the most phenotypic variation) to precisely determine groups of loci that explained the most phenotypic variation or had the lowest classification error rate. Loci identified by backward purging were deemed to be predictive of the phenotypes (i.e., trait‐associated loci).

### 2.6 Gene annotation

As there is no reference genome for Chinook salmon, functions of genes near trait‐associated loci were determined by first aligning loci to the rainbow trout genome (Berthelot et al., 2014) using *Bowtie 2* (v. 2.2.9; Langmead & Salzberg, 2012) with default parameters. After file format conversion, the *closest* function in *bedtools* (v. 2.26.0; Quinlan & Hall, 2010) was then used to identify the rainbow trout gene that was closest to each aligned locus. Coding sequences of genes were then aligned against the UniProtKB/Swiss‐Prot database using a BLASTx search (Altschul, Gish, Miller, Myers, & Lipman, 1990) with default parameter settings and an *e*‐value threshold of 1 × 10^−10^. The search identified UniProtKB/Swiss‐Prot entry names and protein products associated with each gene. Gene Ontology (GO) and GO Slim terms were then identified for each entry name; this approach assumes shared functionality across species. GO Slim terms related to biological processes were summarized to provide biological context for trait‐associated loci. However, to ensure the quality of summaries, GO Slim terms were only used for loci that aligned to the rainbow trout genome with a mapping quality ≥10 and were within 100 kb of a gene. In addition, we retained all GO Slim terms for each protein but, if the same term appeared multiple times for a protein, we counted the term only once to avoid overrepresentation of individual proteins in annotation summaries.

### 2.7 Phenotypic differences between hatchery lines

Phenotypic differences between the integrated and segregated lines were quantified because they may provide the first indication of genetic change at trait‐associated loci. For example, divergence at trait‐associated loci may be more likely if the two lines also exhibit large phenotypic differences. Such divergence may indicate the action of domestication selection since the two hatchery lines experience different levels of exposure to the hatchery. Here, differences were quantified using linear or generalized linear models in R (R Core Team 2016). Return timing, spawn timing, fork length, and daily growth coefficient were modeled with a Gaussian distribution and an identity link function, while age at maturity was modeled using a Poisson distribution and a log link function. The general model form for each of these traits was:

(2) $$y_{i}=\beta_{0}+\beta_{1}{\text{Line}}_{i}+\beta_{2}{\text{Sex}}_{i}+\beta_{3}{\text{Line}}_{i}\times {\text{Sex}}_{i}+e_{i}$$

where *y*_*i*_ was the trait measurement, Line_*i*_ was the hatchery line, and Sex_*i*_ was the sex for individual *i*, respectively. The model for weight comprised the same explanatory variables as (2) but also accounted for allometric growth (e.g., Thorson, 2015) by including fork length in the form of:

(3) $$\text{log}\left(w_{i}\right)=\beta_{0}+\beta_{1}{\text{Line}}_{i}+\beta_{2}{\text{Sex}}_{i}+\beta_{3}{\text{Line}}_{i}\times {\text{Sex}}_{i}+\beta_{4}\text{log}\left(\text{fork length}\right)+e_{i}$$

Divergence between the lines, if present, would be more likely to occur in recent years after multiple generations of hatchery propagation. Therefore, models were analyzed using only measurements from 2010 (*F*_3_ generation), which was the most recent year for which relatively large sample sizes (>50) existed for all traits. Models for four traits—age at maturity, return timing, fork length, and weight—were analyzed using measurements collected from all fish that returned to RAMF in 2010. Spawn timing and daily growth coefficient were analyzed using fish sampled for RAD sequencing, as these traits were only measured in the subset of adults used as broodstock. With the exception of age at maturity, traits were analyzed using only 4‐year‐old fish because they represent >75% of all adults (Knudsen et al., 2006).

### 2.8 Effectiveness of managed gene flow and traits affected by domestication selection

Inferences regarding the effectiveness of managed gene flow to limit divergence at trait‐associated loci, as well as specific traits potentially affected by domestication selection, were made using two comparative methods. First, genetic variation at trait‐associated loci identified by random forest was compared between the integrated and segregated hatchery lines across four generations using principal components analyses (PCA). Temporal divergence of the segregated line from the integrated line in multivariate space, possibly resulting from domestication selection, would suggest that managed gene flow limited change at trait‐associated loci.

Second, trait‐associated loci were compared to loci and genomic regions that were previously identified from the same samples as exhibiting signals of adaptive divergence between the integrated and segregated hatchery lines (i.e., outlier loci and regions; Waters et al., 2015, 2017). Outlier loci and regions had been identified using three tests—*F*_TEMP_ (Therkildsen et al., 2013), *Bayescan* (Foll & Gaggiotti, 2008), and the sliding window approach employed by Hohenlohe et al. (2010). *F*_TEMP_ detects selection in a single population sampled across multiple generations by simulating genetic drift over time and identifying loci that exceed neutral expectations; we conducted *F*_TEMP_ separately for each hatchery line to identify outliers. *Bayescan* estimates population‐ and locus‐specific components of *F*_ST_ for each locus and identifies outliers when the locus‐specific component is significantly different from zero; due to the small number of populations in our analysis, this program was run with both hatchery lines combined and thus identified a single set of outliers. The sliding window approach randomly samples empirical *F*_ST_ values from all mapped study loci to generate a null distribution reflecting genomewide divergence due to neutral processes. Outlier regions were identified for each generation of each hatchery line as those where the moving average of *F*_ST_ (compared to the *P*_1_ Founders) exceeded the 95% confidence interval of the null distribution. Overlap between outliers identified by these three methods and trait‐associated loci was interpreted as evidence that specific traits had responded to domestication selection.

## 3 Results

### 3.1 Tissue sample and phenotypic data collection

Tissues of 753 returning adult Chinook salmon, representing the 1998 wild founders and four generations of each hatchery line, were subsampled (Table S1). Phenotypic traits (Table 1) for these and other adults that returned to the upper Yakima River were measured at RAMF (Table S2a,b). For fish used as broodstock, phenotypic data were also recorded upon spawning at CESRF (Table S2b).

### 3.2 DNA sequencing and genotyping

DNA from 696 of the 753 tissue samples was successfully sequenced, and 11809 biallelic loci were identified using *Stacks*. Re‐genotyping of these loci using the custom Python script of Brieuc et al. (2014) improved the distribution of *F*_IS_ values based on Hardy–Weinberg expectations (e.g., median *F*_IS_ across loci for *P*_1_ Founders was approximately 0.3 and 0.1 before and after the custom script, respectively). Filtering based on minor allele frequency and missing genotypes reduced the data set to 465 individuals and 9,266 loci. Missing genotypes were then imputed for random forest analyses; the correlation of allele frequencies at each locus before and after imputation was 0.998 (Figure S1), suggesting that imputation did not significantly compromise the data. Genotypes at 158 loci significantly deviated from expected Hardy–Weinberg proportions in more than one population and were removed. Of these 158 loci, 103 and 125 significantly deviated from Hardy–Weinberg equilibrium in the *P*_1_ Founders and *F*_1_ Wild populations, respectively. These samples represent pure wild fish (i.e., no exposure to hatchery conditions); thus, deviations at these loci in these populations occurred from factors other than domestication selection. The final data set comprised 465 individuals genotyped at 9,108 loci (Tables S3 and S4).

### 3.3 Inferring positions of unmapped loci

The 9,108 study loci included 4,156 mapped loci and 4,952 unmapped loci. Of the unmapped loci, 616 and 198 aligned to the rainbow trout and Atlantic salmon genomes, respectively, with mapping qualities ≥10 and were within 100 kb of a mapped locus. After accounting for those that aligned to both genomes, 700 unmapped loci were assigned the same linkage map positions as their corresponding mapped loci (Tables S3 and S5). Therefore, the positions of 4,856 loci were known for downstream analyses.

### 3.4 Random forest analyses

The number of individuals analyzed by random forest varied slightly for each trait (Table 2), as those with missing phenotypes were removed prior to analyses. For age at maturity, sample sizes of three‐, four‐, and five‐year‐old fish were 23, 344, and 16, respectively; the use of balanced classification trees accounted for this unequal distribution. The number of trees needed to obtain high correlation (>0.8) of importance values between initial forests varied from 250,000 to 750,000 for the traits.

**Table 2:** Results of random forest association analyses for six phenotypic traits. For each trait, the number of individuals analyzed, the total number of predictor loci identified by the backward purging approach and the number of those that were mapped, and the percent trait variation explained by the predictor loci are given

Trait	Individuals	Predictor loci (mapped)	Percent variation explained
Age at maturity	383	30 (20)	24.9^a^
Return timing	383	26 (15)	29.2
Spawn timing	379	68 (36)	26.7
Fork length	380	44 (20)	32.0
Weight	381	37 (15)	31.5
Daily growth coefficient	372	35 (20)	31.8

a Denotes the out‐of‐bag classification error rate for age at maturity, rather than variation explained, as the trait was analyzed using classification trees. Here, classification error rate refers to the percentage of hold‐out samples whose ages were misclassified using the tree.

The backward purging approach identified 226 unique predictor (i.e., trait‐associated) loci over all traits, with 53% having known or inferred positions on the Chinook salmon linkage map (Table 2; Table S6). The number of predictor loci per trait ranged from 26 to 68. All predictors for each of the five continuous traits explained 26.7% to 32.0% of the observed phenotypic variation, while loci predictive of age at maturity had an out‐of‐bag classification error rate of 24.9% (i.e., rate at which the hold‐out samples are misclassified using the random forest model; Table 2).

**Figure 2:**
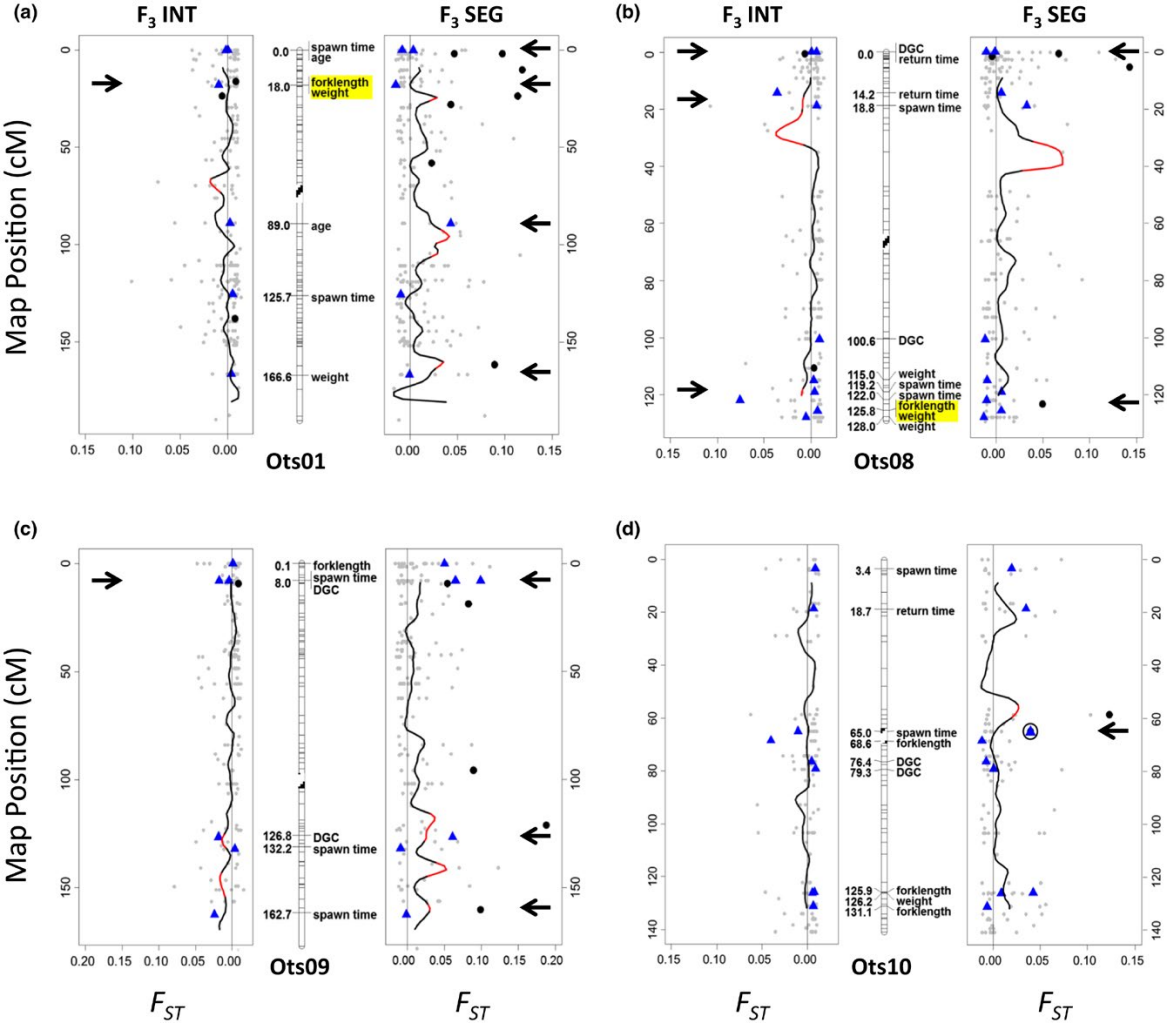
Graphical representation of four Chinook salmon chromosomes (center panels, a–d) showing the map positions (cM) of loci associated with six fitness‐related traits, as identified by random forest analyses. Loci associated with different traits mapped to the same regions, including loci on Ots01 and Ots08 that were associated with both fork length and weight (highlighted in yellow). Divergence (*F*_ST_) of the *F*_3_ INT and *F*_3_ SEG hatchery lines when compared to the *P*_1_ founders is displayed in the left and right panels of each figure, respectively. The *F*_3_ generation is shown because it is the most recent hatchery generation for which there are relatively large sample sizes (>50), and thus has greater statistical support for all outlier tests. The black line denotes the moving average of *F*_ST_ across the chromosome, with regions exhibiting significant levels of divergence (i.e., outlier regions) from the *P*_1_ Founders in red (Waters et al., 2015, 2017). The centromere of each chromosome is shaded with diagonal black lines. Black circles represent outlier loci previously identified by *F*_TEMP_ and *Bayescan*, blue triangles correspond to trait‐associated loci, and gray points are all other study loci. Locations where trait‐associated loci are in close proximity to outlier loci or regions are marked with black arrows, including one outlier locus on Ots10 that was also associated with spawn timing (circled)

Trait‐associated loci were identified on 33 of the 34 chromosomes for Chinook salmon (Figure S2; Table S6). Notably, some loci were associated with multiple traits. For example, 12 loci were identified as predictors for both fork length and weight at return (e.g., Figures 2a, b). In addition, one locus was associated with both daily growth coefficient and return timing on chromosome Ots21, while an unmapped locus was shared between fork length and spawn timing. Other predictor loci did not overlap but still mapped to the same genomic regions, both within and across traits. For instance, two loci associated with daily growth coefficient were within 3 cM of each other on Ots10 (Figure 2d), and two loci predictive of spawn timing mapped to the same position on Ots31. Two loci for weight, two for spawn timing, and one for both fork length and weight all mapped to a 13 cM region on chromosome Ots08 (Figure 2b). Similarly, two loci associated with fork length and one locus associated with weight mapped to a 5 cM region on chromosome Ots10 (Figure 2d). Two maturation‐related traits, spawn timing and age at maturity, had predictor loci within 0.01 cM of each other on three chromosomes (Ots01, Ots02, Ots26; Figure 2a). Loci for daily growth coefficient and return timing mapped to the same position on chromosome Ots08 (Figure 2b), while loci for daily growth coefficient and spawn timing mapped to similar positions on Ots09 (Figure 2c). Lastly, two narrow regions on Ots04 and Ots19 (same position and 2.2 cM wide, respectively) each contained one locus associated with size (fork length or weight) and one for daily growth coefficient.

### 3.5 Gene annotation

Annotations for 75 of the 226 trait‐associated loci were obtained after filtering for loci that aligned to the rainbow trout genome with a mapping quality ≥10 and were within 100 kb of a gene (Table S7a). Sixty‐three of these genes had GO Slim terms associated with biological processes, the functions of which varied within and between traits. The biological processes represented by the most genes across traits included cell organization and biogenesis, developmental processes, transport, and other biological processes (Table S7b).

The low annotation rate limited exploration of gene functions for genomic regions where trait‐associated loci overlapped. For example, only two of the 12 loci identified as predictors for both fork length and weight were annotated and met quality thresholds. There were, however, a few regions of interest. The *intersectin 2* (ITSN2) gene, which is involved in cell membrane transport, was only 300 base pairs from two loci associated with return timing and daily growth coefficient that mapped to the same position on Ots08 (Figure 2b). The two loci linked to daily growth coefficient on Ots10 (Figure 2d) were within and near (13 kb) the *sin3A associated protein 130* (SAP130) and *prostaglandin‐endoperoxide synthase 2* (PTGS2) genes, respectively, which are involved in transcription and stress response. Another region on Ots10 contained predictors for fork length and weight (Figure 2d); one of these loci was within a gene that codes for phosphatase and actin regulator 1, a protein involved in cell regulation. Loci for spawn timing and age at maturity mapped to the same position on Ots02; one of the predictors was within the *ADP ribosylation factor like GTPase 6 interacting protein 5* (ARL6IP5) gene, which is involved in membrane traffic. Lastly, of the two co‐mapping loci related to spawn timing on Ots31, one was within the *mitogen‐activated protein kinase 15* (MAPK15) gene, which is involved in signal transduction and DNA, RNA, and protein metabolism.

### 3.6 Phenotypic differences between hatchery lines

After multiple generations of rearing, the effect of hatchery line on phenotype was evident for some but not all traits (Table 3). In the *F*_3_ generation, 4‐year‐old fish from the two lines significantly differed in weight at return, spawn timing, and daily growth coefficient. Specifically, fish from the segregated line weighed less (at a given length) than fish from the integrated line, their gonads matured earlier, and they had larger daily growth coefficients (i.e., they lost less weight during holding at the hatchery than fish from the integrated line; Table 3). The interaction between line and sex was significant for return timing and daily growth coefficient, indicating that the effect of hatchery line differed between sexes for these traits. Age at maturity, fork length at return, and the main effect of return timing were not different between the lines (Table 3).

**Table 3:** Sample sizes from each hatchery line, regression coefficients (standard errors) of terms, and test statistics with *p*‐values based on linear or generalized linear models for each phenotypic trait. Significant *p*‐values are in bold font. The reference level for the intercept is integrated females. Due to the form of the models, coefficients for age at maturity and weight were exponentiated (except for β_fork length_) and refer to the proportionate response compared to the reference level. β_fork length_ is the allometric coefficient

	Age at maturity (years)	Return timing (day of year)	Fork length (cm)	Weight (kg)	Spawn timing (day of year)	DGC
*N*_INT_	1135	984	984	984	60	60
*N*_SEG_	247	200	200	200	52	48
β_Intercept_	4.00 (0.078) *Z *=* *71.39 ***p *** **<** *** *** **.001**	156.57 (0.77) *t *=* *204.42 ***p *** **<** *** *** **.001**	72.34 (0.17) *t *=* *425.42 ***p *** **<** *** *** **.001**	1.28e^−05^ (1.98e^−06^)*t* = −77.85 ***p *** **<** *** *** **.001**	265.75 (1.01) *t *=* *263.78 ***p *** **<** *** *** **.001**	−0.05 (0.003) *t *=* *−16.49 ***p *** **<** *** *** **.001**
β_Line_	1.00 (0.047) *Z *=* *0.00 *p *=* *1.000	3.35 (1.86) *t *=* *1.80 *p *=* *.072	−0.70 (0.41) *t *=* *−1.70 *p *=* *.089	0.98 (0.01) *t *=* *−2.54 ***p *** **=** *** *** **.011**	−9.26 (1.36) *t *=* *−6.81 ***p *** **<** *** *** **.001**	0.01 (0.004) *t *=* *2.59 ***p *** **=** *** *** **.011**
β_Sex_	0.92 (0.028) *Z *=* *−2.56 ***p *** **=** *** *** **.011**	−0.87 (1.33) *t *=* *−0.65 *p *=* *.514	0.44 (0.30) *t *=* *1.50 *p *=* *.135	0.99 (0.005) *t *=* *−1.49 *p *=* *.138	−1.46 (1.47) *t *=* *−0.99 *p *=* *.323	−0.03 (0.004) *t *=* *−5.87 ***p *** **<** *** *** **.001**
β_Line*Sex_	0.98 (0.071) *Z *=* *−0.31 *p *=* *.760	7.34 (3.25) *t *=* *2.25 ***p *** **=** *** *** **.024**	0.74 (0.72) *t *=* *1.02 *p *=* *.31	0.98 (0.01) *t *=* *−1.42 *p *=* *.157	−0.79 (2.35) *t *=* *−0.34 *p *=* *.736	0.02 (0.007) *t *=* *2.24 ***p *** **=** *** *** **.027**
β_fork length_	N/A	N/A	N/A	2.98 (0.03) *t *=* *88.02 ***p < *** **.001**	N/A	N/A

Phenotypic differences between the two hatchery lines were also present in other generations but varied (data not shown). Return timing, fork length, and weight significantly differed between the lines in the *F*_1_ generation, while spawn timing differed in the *F*_2_ generation. The interaction of hatchery line and sex was significant for fork length in the *F*_4_ generation.

### 3.7 Effectiveness of managed gene flow and traits affected by domestication selection

#### 3.7.1 Comparisons across all trait‐associated loci

Evaluations of genetic variation at trait‐associated loci using PCA showed little evidence of divergence between the hatchery lines across all traits. Each generation of the two lines overlapped extensively in multivariate space at loci associated with traits that showed significant phenotypic differentiation (spawn timing, daily growth coefficient, and weight; Figures S3–S5; Table S10) and those that did not (return timing, age at maturity, and fork length; Figures S6–S8; Table S10).

#### 3.7.2 Overlap with outlier loci and outlier regions

**Figure 3:**
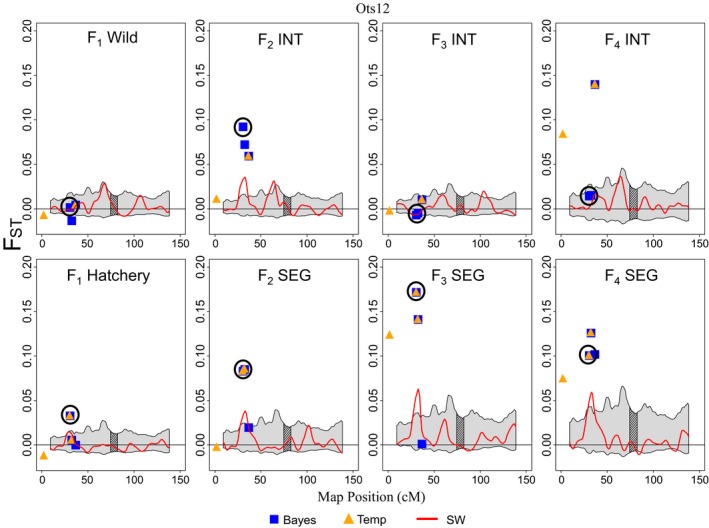
Loci and regions of chromosome Ots12 showing signatures of adaptive divergence based on measures of pairwise *F*_ST_ between each generation of each line and the *P*_1_ founders. The results are given for the integrated (top panel) and segregated (bottom panel) hatchery lines through the *F*_1_, *F*_2_, *F*_3_, and *F*_4_ generations. Blue squares are loci that were identified as outliers by *Bayescan* (Foll & Gaggiotti, 2008) and orange triangles are outliers identified by *F*_TEMP_, a method designed to detect selection in a single population over time (Therkildsen et al., 2013). The red line represents the kernel‐smoothed moving average of *F*_ST_, and the gray shaded area is the 95% confidence interval. Genomic regions exhibiting significant levels of divergence (i.e., outlier regions) from the *P*_1_ founders occur where the moving average of *F*_ST_ exceeds the 95% confidence intervals. The centromere of the chromosome is shaded with diagonal black lines. The black circle designates a locus predictive of return timing, Ot005185_Ots12p, which was also identified as an outlier by *Bayescan* and, in the segregated line, by *F*_TEMP_. Negative *F*_ST_ values occur due to finite sample sizes and slight variance in sample sizes between populations (Weir & Cockerham, 1984)

Comparisons between trait‐associated loci and previously identified outlier loci (Waters et al., 2015, 2017) revealed six loci that overlapped (Table S8a). Specifically, four loci associated with spawn timing had also been identified as outliers unique to the segregated hatchery line. In addition, one locus linked to return timing on Ots12 was identified as an outlier by *Bayescan* and, in the segregated line, by *F*_TEMP_ (Figure 3). This locus was near two other outliers and was located within a region that exhibited significant divergence from the *P*_1_ founders across all four generations in the segregated line (Figure 3). The region was also significantly divergent in the *F*_2_ generation of the integrated line. Lastly, one unmapped locus was associated with both fork length and weight and was identified as an outlier by *Bayescan* and *F*_TEMP_ in both hatchery lines.

Regions of interest were also designated where trait‐associated loci were in close proximity to outlier loci and regions (Tables S8b,c). Six regions in the segregated line, for example, exhibited significantly elevated divergence from the *P*_1_ founders in at least one generation and also contained both trait‐associated and outlier loci (located on Ots01, two regions on Ots09, Ots11, Ots12, Ots30; Table S8c; Figures S9 and S11). The integrated line contained three such regions (located on Ots12, Ots15, Ots30; Table S8b; Figures S10 and S11), with those on Ots12 and Ots30 coinciding with the segregated line. Other chromosomes also contained regions where trait‐associated loci and outliers were in close proximity (e.g., black arrows, Figure 2), although the overlap was not as extensive.

Gene annotations provided functional insight for some of these loci and regions of overlap (Tables S7a,b and S8a–d). One locus on Ots10 that was both an outlier and associated with spawn timing, for example, was located near a second outlier locus and an outlier region in the segregated line (Figure 2d). The nearby outlier locus was 800 base pairs from the *transcriptional adaptor 2B* (TADA2B) gene, which aids in transcription (Table S8d). The region of overlap on Ots01, which displayed elevated divergence in the *F*_4_ SEG generation, contained a locus associated with weight (position 166.6 cM; Figure 2a) and one outlier locus. The locus linked to weight was within a gene that codes for tyrosine‐protein kinase, which has many functions, while the outlier locus was near a gene related to developmental processes, cell organization and biogenesis, and transport. Loci within the region of overlap on Ots12 (Figure 3) were in genes related to signal transduction, cellular protein modification, cell morphogenesis, kinase activity, and developmental processes (Table S8d; Waters et al., 2015). The region on Ots15 (Figure S10) contained a locus linked to fork length and two outlier loci. The trait‐associated locus was 2 kb from the *slit‐robo rho GTPase activating protein 1* (SRGAP1) gene, which is related to signal transduction and other biological and metabolic processes. One of the outlier loci was also annotated; it was 13 kb from the *member RAS oncogene family* (RAB32) gene, which plays a role in cell organization and biogenesis, transport, signal transduction, and other biological and metabolic processes. Finally, the segment on Ots30 contained a locus associated with fork length, a locus predictive of age at maturity, and a locus that had been identified as an outlier by *Bayescan* and *F*_TEMP_ in the segregated line (Figure S11). The locus associated with fork length was within the *cut like homeobox 1* (CUX1) gene, which is involved in developmental processes, RNA metabolism, and cell organization and biogenesis. No other loci from this region could be annotated.

## 4 DISCUSSION

Here, we aimed to identify genomic regions that influence six fitness‐related traits in adult Chinook salmon that are captive‐reared but spend part of their life cycle in the natural environment. We then explored the use of trait‐associated loci within a management context; namely, whether they could serve as tools for monitoring the effects of two different hatchery management approaches on genetic change in phenotypic traits across four generations. The integrated line uses only wild‐born broodstock, and all hatchery‐born fish from this line are allowed to spawn in the wild. In contrast, the segregated line uses only hatchery‐born broodstock, and all hatchery‐born fish from this line are removed from the river before reproduction. By comparing these lines, our study is one of the first to utilize genomic approaches to determine the effectiveness of a conservation strategy, managed gene flow, on trait‐associated—and potentially adaptive—loci.

We detected 226 unique loci associated with the six traits. Some loci were associated with multiple traits while others mapped to shared positions on the genome, results that may be due to the fact that many fitness traits are phenotypically and genetically correlated (Carlson & Seamons, 2008; Hard, 2004). No evidence for broad‐scale genetic change at trait‐associated loci was observed between the integrated and segregated hatchery lines across four generations using PCA. However, numerous regions were identified where trait‐associated loci overlapped with outliers. Many of these overlapping regions, primarily with loci linked to spawn timing and return timing (e.g., Figure 2d and 3), were either unique to, or more divergent in, the segregated hatchery line. Other regions were present in both hatchery lines (e.g., fork length, Figure S11). These results highlight the role that managed gene flow plays in reducing genetic change at loci linked to important phenotypic traits. Continued monitoring of these loci will provide further insights into processes influencing polygenic traits in captive populations.

### 4.1 Detecting trait‐associated loci in natural populations

A key challenge in applying genomic‐based approaches to conservation is the ability to detect loci linked to polygenic traits in natural populations (Olson‐Manning, Wagner, & Mitchell‐Olds, 2012; Pritchard & Di Rienzo, 2010). Notably, experimental power to detect trait‐associated loci may be limited by sample size, marker coverage across the genome, and effect sizes of loci on the traits of interest (Korte & Farlow, 2013). While the current study may have been limited by such factors, the data set analyzed here is typical for natural populations and comparable with those from other recent association studies with an evolutionary emphasis (Brieuc et al., 2015; Hess et al., 2016; Laporte et al., 2015; Nichols et al., 2016; Pavey et al., 2015). In addition, random forest is predicted to perform well in natural populations when linkage disequilibrium between quantitative trait loci and study markers may be reduced (Holliday et al., 2012) or sample size is small (Rokach, 2016), which are typical conditions for many conservation scenarios.

Power to detect genotype–phenotype associations may also have been reduced by the amount of phenotypic variation within the six traits that were analyzed, as the study was restricted to variability found within a single river system. For example, the standard deviations of return timing and spawn timing across all individuals were 22 and 8 days, respectively (Table S2b). Similarly, over 75% of the adult Chinook salmon in the study population mature at age four (Knudsen et al., 2006), leaving few mature adults of other ages. In contrast, other studies employing random forest have identified associations in traits with greater variance (e.g., seasonal salmonid migration timing; Brieuc et al., 2015; Hess et al., 2016). Incorporating other populations of Chinook salmon for which extensive genetic and phenotypic data exist, though rare, will improve our ability to identify the genetic basis of key traits in the future.

This study is one of the first to use the same genomic dataset to analyze associations with several covarying traits in adult Chinook salmon. In doing so, we observed that the percent phenotypic variation explained by random forest was similar across all of the traits (25%–32%), despite the fact that the number of predictor loci varied for each trait. The similarity of the results is likely due to the power of the study design. Each tree in random forest is grown to its maximal depth, or until a designated stopping criterion is reached (Goldstein, Polley, & Briggs, 2011; Liaw & Wiener, 2002). The depth of the trees and the predictive power of the algorithm are partially dependent on the number of individuals and number of loci analyzed, which were nearly identical across traits. Likewise, the number of individuals analyzed directly influences the calculation of percent variation explained (Liaw & Wiener, 2002). Therefore, the similar percentages of variance explained across traits may reflect the size of the study. Nevertheless, we considered the designation of predictor loci as preliminary and placed an emphasis on loci that were verified with additional evidence.

### 4.2 Mapping and annotation of trait‐associated loci

Further support for candidate loci identified by random forest was obtained by comparing the genome map positions of loci across all fitness‐related traits. Specifically, we interpreted sites that contained multiple predictors, including loci that were associated with more than one trait, as candidates for regions underlying fitness. For instance, the 12 loci that were associated with both fork length and weight, traits that are phenotypically (Pearson's *r* = .92 for fork length and weight in this study) and genetically correlated in Chinook salmon (Carlson & Seamons, 2008; Hard, 2004), may impact growth and, in turn, survival. One of these loci was 875 base pairs from a gene involved in microtubule bundle formation, while another locus was 5 kb from the *DnaJ heat shock protein family (Hsp40) member C5* (DNAJC5) gene, which regulates the exocytosis of insulin (Lang, 1999), a hormone critical for processing glucose. The 13 cM region on Ots08 and the 5 cM segment on Ots10, which each contained three loci linked to length or weight, may also affect metabolic processes and growth. One locus from the region on Ots10 was within *phosphatase and actin regulator 1* (PHACTR1), which is a key regulator of endothelial cells (Jarray et al., 2011) and is also significantly associated with coronary artery calcification in humans (van Setten et al., 2013).

Similarly, regions that may be linked to maturation were identified on Ots31, where two loci associated with spawn timing mapped to the same position, and on three other chromosomes (Ots01, Ots02, Ots26), which each had predictor loci for spawn timing and age at maturity within 0.1 cM of each other. The two annotated loci from these regions were within the ARL6IP5 and MAPK15 genes on Ots02 and Ots31, respectively. ARL6IP1, a gene that associates with ARL6IP5, affects neurotransmission through Na+‐dependent neural glutamate transport activity (Akiduki & Ikemoto, 2008). ARL6IP5 also positively regulates the MAPK pathway (Safran et al., 2010), to which MAPK15 belongs. The MAPK pathway regulates numerous processes, including cell growth, differentiation, and apoptosis (Qi & Elion, 2005), and can be activated by stressful conditions such as heat shock (Dorion & Landry, 2002). The association of these two genes with maturation traits in salmon may be related to the transition from marine to freshwater environments that occurs prior to spawning (change in Na+ concentration) and its concomitant stressors (e.g., cessation of feeding, exposure to warmer temperatures, physiological costs associated with upriver migration, development of secondary sex characteristics, and senescence).

Lastly, regions containing multiple loci associated with return timing or daily growth coefficient (e.g., Ots08, Figure 2b; 3 cM segment on Ots10, Figure 2d) may also be involved in the marine to freshwater transition and maturation process. Annotations of these regions identified the ITSN2 (Ots08), SAP130 (Ots10), and PTGS2 (Ots10) genes as potential candidates. ITSN2 regulates cell membrane traffic via clathrin‐mediated endocytosis (Pucharcos, Estivill, & de la Luna, 2000), SAP130 is a transcriptional repressor (Fleischer, Yun, & Ayer, 2003), and PTGS2 expression is related to inflammation, blood vessel formation, estrogen synthesis, and reduced apoptosis (Marshall et al., 2005). However, the functional importance of these and other regions requires further exploration and will improve with the development of additional genomic resources for Chinook salmon.

### 4.3 Comparative analyses across studies

Comparison of our results to those from other studies of Chinook salmon provided additional evidence for certain genomic regions underlying fitness. Notably, a circadian clock gene that was divergent between spring‐ and fall‐migrating populations of Chinook (OmyFbxw11; O'Malley, Jacobson, Kurth, Dill, & Banks, 2013) was 2 cM from the region on Ots08 in which we identified five predictor loci for spawn timing, length, and weight (Figure 2b; Table S9). Similarly, Brieuc et al. (2015) identified two predictors of adult seasonal migration time on Ots12 and Ots17 that were 2.3 cM and 0.6 cM, respectively, from loci that we linked to spawn timing (Table S9). A locus that we linked to fork length on Ots10 was in the same 3 cM region where Brieuc et al. (2015) identified five predictors of migration time. Lastly, two thermotolerance QTL (quantitative trait loci; Everett & Seeb, 2014; McKinney et al., 2016) mapped to the same positions as loci associated with age at maturity and return timing on Ots11 and Ots27, respectively. Migration and spawn timings in Chinook salmon are known to exhibit strong phenotypic and genetic correlations in some populations (e.g., Quinn, Unwin, & Kinnison, 2000), and body size may also be correlated with these traits as it can influence access to spawning grounds and breeding success (Lin et al., 2016; Schroder et al., 2008). The colocation of thermotolerance QTL with loci linked to age and return timing is also supportive, as the migration of spring Chinook salmon is correlated with water temperature (Keefer, Peery, & Caudill, 2008). These regions should be specifically targeted by future investigations that aim to identify the specific genes underlying fitness‐related traits.

### 4.4 Traits affected by domestication selection

Although significant phenotypic differences were observed between the integrated and segregated hatchery lines for weight, spawn timing, and daily growth coefficient, no trait exhibited genetic change over time when all associated loci were examined using PCA. This result may be due to the effectiveness of recent hatchery reforms (Mobrand et al., 2005; Paquet et al., 2011) and the management practices of CESRF (Fast et al., 2015), which aim to minimize potential negative ecological and genetic effects of captive rearing. However, null results may also reflect insufficient power to detect genetic divergence at trait‐associated loci. Adaptation of complex quantitative traits likely involves selection on standing genetic variation (Bernatchez, 2016; Fu & Akey, 2013) and is predicted to result in minor allele frequency changes at many loci, or an increased degree of covariance across loci (Le Corre & Kremer, 2012; Pritchard & Di Rienzo, 2010). Therefore, domestication selection may not produce discernible genetic change across many trait‐associated loci, particularly after just four generations of captive rearing.

Yet, specific candidates for where domestication selection may be affecting variation underlying certain traits were identified through comparisons between trait‐associated loci and signatures of adaptive divergence from previous studies (Waters et al., 2015, 2017). Spawn timing, for example, is a fitness trait that exhibited significant phenotypic divergence between the two hatchery lines and also showed the most overlap between trait‐associated and outlier loci (four loci). Each of these four overlapping loci was an outlier in the segregated line, which is exposed to hatchery conditions every generation, but not in the integrated line, which is only exposed to the hatchery for one generation (Figure 2d; Table S8a). There was also a fifth locus associated with spawn timing on Ots09 that was located within an outlier region in the *F*_3_ and *F*_4_ generations of the segregated line but not in the integrated line (Tables S8b,c). Similarly, phenotypic comparisons of return timing revealed a significant interaction of hatchery line and sex (Table 3), suggesting that hatchery rearing may be disproportionately affecting this trait in males than females, although the mechanisms of such an effect remain unclear. Genetic comparisons identified one locus linked to return timing on Ots12 that exhibited signals of adaptive divergence by *Bayescan* and, in the segregated line, by *F*_TEMP_ (Figure 3). Notably, divergence of the region containing this locus was consistent across all four generations of the segregated line, compared to just one generation in the integrated line.

It should be noted that the greater levels of overlap observed between trait‐associated and outlier loci in the segregated line may, in part, be due to an increased ability to detect outliers in that line, which has a smaller effective population size (Waters et al., 2015, 2017) and thus potentially higher levels of linkage disequilibrium between study loci and loci under selection. In addition, this study lacks a wild “control” population and thus cannot fully discriminate between processes that occur in the hatchery and those that occur in the natural environment, such as natural selection. Nevertheless, multiple lines of evidence—phenotypic divergence, greater overlap with outliers in the segregated line than in the integrated line, and temporal consistency—suggest that these regions may be responding to domestication selection on return and spawn timing. Our results also support those from other systems, where phenotypic differences in return and spawn timing between wild and hatchery‐reared Chinook salmon have been observed (Hoffnagle et al., 2008; Williamson, Murdoch, Pearsons, Ward, & Ford, 2010).

### 4.5 Conclusions and conservation implications

The utility of trait‐linked markers in conservation genetics is being actively discussed and explored (Bernatchez et al., 2017; Garner et al., 2016; Pearse, 2016; Shafer et al., 2015). For example, markers associated with key phenotypic traits can provide further insights into the mechanisms by which adaptive variation is maintained in populations (Bernatchez, 2016) and may also assist the delineation of conservation (Brieuc et al., 2015; Funk et al., 2012; Garner et al., 2016) and fisheries management units (Bernatchez et al., 2017). This study applied trait‐associated loci in a novel way—to evaluate the effects of alternative management approaches in captive breeding on genetic variation underlying several fitness traits. Our findings demonstrate the future utility of genomic‐based approaches in conservation monitoring.

The identification of loci associated with six key traits by random forest is a first step toward characterizing the functional genetic basis of fitness in Chinook salmon (Macqueen et al., 2017). The trait‐associated loci were supported by genome mapping, gene annotations, and the integration of results across multiple studies. In the future, these loci may contribute to the development of trait‐specific tools to monitor genetic change in captive and wild populations, and to better understand the responses of populations to conservation actions and environmental variability (e.g., climate change). The regions where trait‐associated and outlier loci overlapped will provide useful starting points for future sequencing efforts that aim to identify the specific genes responding to domestication selection. The observed phenotypic and genomic divergence in certain traits, most notably spawn timing, may also have more immediate impacts on specific management practices at CESRF to further reduce possible effects of captive rearing, as the program is adaptively managed (Fast et al., 2015). Lastly, the results support previous work demonstrating the effectiveness of managed gene flow in conservation‐focused breeding programs (Waters et al., 2015, 2017) and will provide additional information that managers can use to assess the relative advantages and disadvantages of different captive rearing approaches.

## DATA ARCHIVING STATEMENT

Raw sequence data for this study are available at the NCBI Sequence Read Archive, accession SRP127673. Sequencing barcodes are provided in Table S11, and genotypes used for analyses are provided in Table S4. Input files and R scripts for random forest analyses and phenotypic comparisons between the hatchery lines have been deposited in the Dryad Digital Repository: https://doi.org/10.5061/dryad.08hc4.
