# Supplementary material for: Genomewide association analyses of fitness traits in captive‐reared Chinook salmon: Applications in evaluating conservation strategies
Source: Evol Appl. 2018 Mar 5;11(6):853–68. doi: 10.1111/eva.12599 (PMC5999212; doi:10.1111/eva.12599)
Supplement: Supplementary file 1 [file EVA-11-853-s001.docx]

Supporting Information for “Genome-wide association analyses of fitness traits in captive-reared Chinook salmon: Applications in evaluating conservation strategies”

*Imputation of missing genotypes*


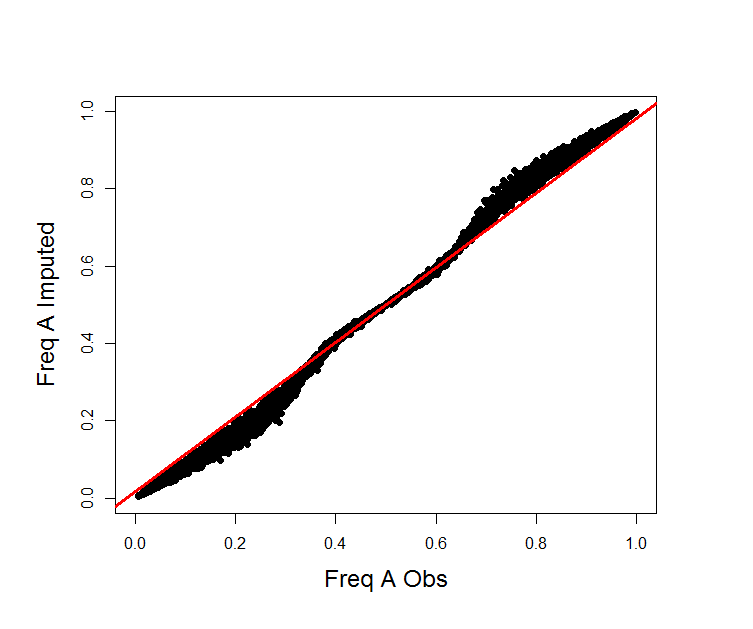
 Missing genotypes were imputed using *fastPhase* (Scheet & Stephens, 2006) because Random Forest cannot handle missing data. Imputation did not alter allele frequencies; the correlation of allele frequencies before and after imputation was 0.998 (Fig. S1).

Figure S1. Plot of allele frequencies (one allele per locus) before (obs) and after imputation of missing genotypes. The correlation of frequencies was 0.998.

*Inferring positions of unmapped loci*

Of the 6350 non-duplicated loci on the Chinook salmon linkage map (Brieuc et al., 2014), 4771 and 3680 loci aligned to the rainbow trout and Atlantic salmon genomes, respectively. The 9108 study loci included 4952 unmapped loci; 3799 and 3103 of these loci aligned to the rainbow trout and Atlantic salmon genomes. However, only 616 and 198 pairs of mapped and unmapped loci that aligned to the rainbow trout and Atlantic salmon genomes, respectively, had mapping qualities >= 10 and were within 100 kb of each other. The unmapped loci from the high quality pairs were assigned the same linkage map position as their corresponding mapped loci (Tables S3, S5). Positions for 113 loci were inferred from both genomes; the positions closely agreed for 112 of the 113 loci. For these loci, positions inferred from rainbow trout were used since rainbow trout are more closely related to Chinook salmon. The one locus for which the two inferred positions did not agree was not assigned a linkage map position. Overall, positions for 700 of the 4952 unmapped loci were inferred (Tables S3, S5).


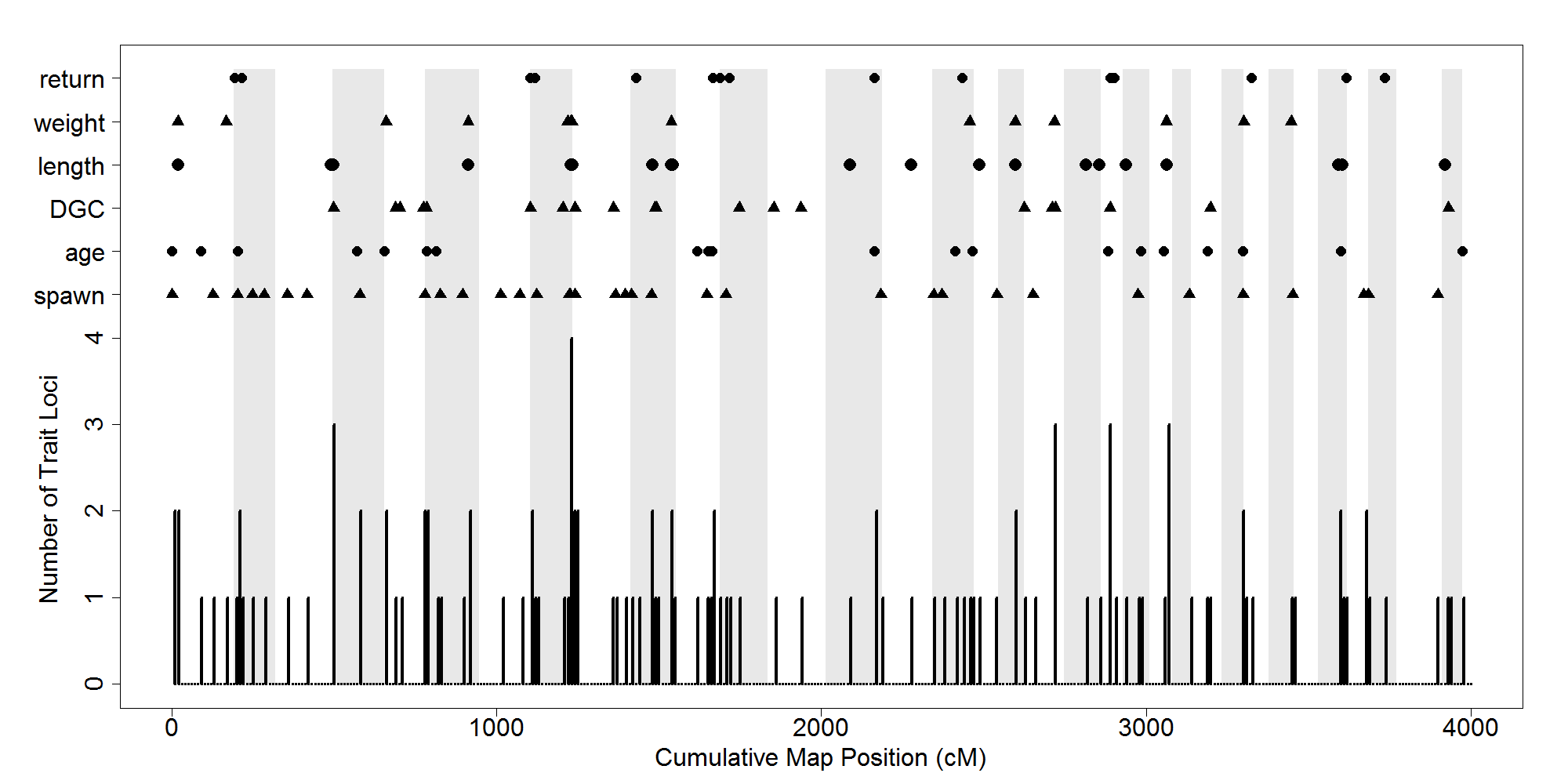
*Random Forest analyses*

Figure S2. Linkage map positions of loci associated with each trait and the number of trait-associated loci within each 10cM bin across the linkage map. Chromosomes Ots01 to Ots34 are shown from left to right, with white and gray backgrounds denoting different chromosomes.

*Gene annotation*

Of the 226 unique predictor loci, 170 aligned to scaffolds of the rainbow trout genome. The *closest* function then identified rainbow trout genes on the same scaffolds for 139 of these loci (Table S7a). Coding sequences for 130 of the 139 rainbow trout genes were successfully aligned against the UniProtKB/Swiss-Prot database, and GO Slim terms were identified for 124 genes (Table S7a). However, annotations for only 75 genes remained after filtering loci for a mapping quality ≥10 and distances within 100kb of a gene. Sixty-three of these genes had GO Slim terms associated with biological processes (Tables S7a,b). Duplicate GO Slim terms within each protein were removed to avoid overrepresentation of individual proteins in annotation summaries.

The functions of annotated genes varied within and between traits. Multiple GO terms that assigned to genes near loci predictive of age at maturity were related to ion and sodium transport (Tables S7a,b), a result that could be due to osmoregulatory changes that occur as salmon mature and transition from marine to freshwater environments. Genes near loci linked to spawn timing were also associated with transport, as well as angiogenesis, the processing of lipids, and a variety of other metabolic processes. These associations may be related to the vascularization of gonads that occurs during maturation and the fact that adult salmon stop feeding during their freshwater migration to the spawning grounds. Some genes near loci associated with daily growth coefficient were linked to the G-protein coupled receptor signaling pathway, which is involved in a variety of biological and cellular processes, including cell adhesion, immune regulation, tumor growth, and metabolic processes (Tables S7a,b). Genes related to return timing, fork length, and weight also had diverse functions (Tables S7a,b).

*Effectiveness of managed gene flow and traits affected by domestication selection*

Principal components analyses were used to visualize temporal changes in genetic relationships between the two hatchery lines at trait-associated loci (as identified by Random Forest; Figs. S3-S8). Loadings of individual loci for each PCA are provided in Table S10.


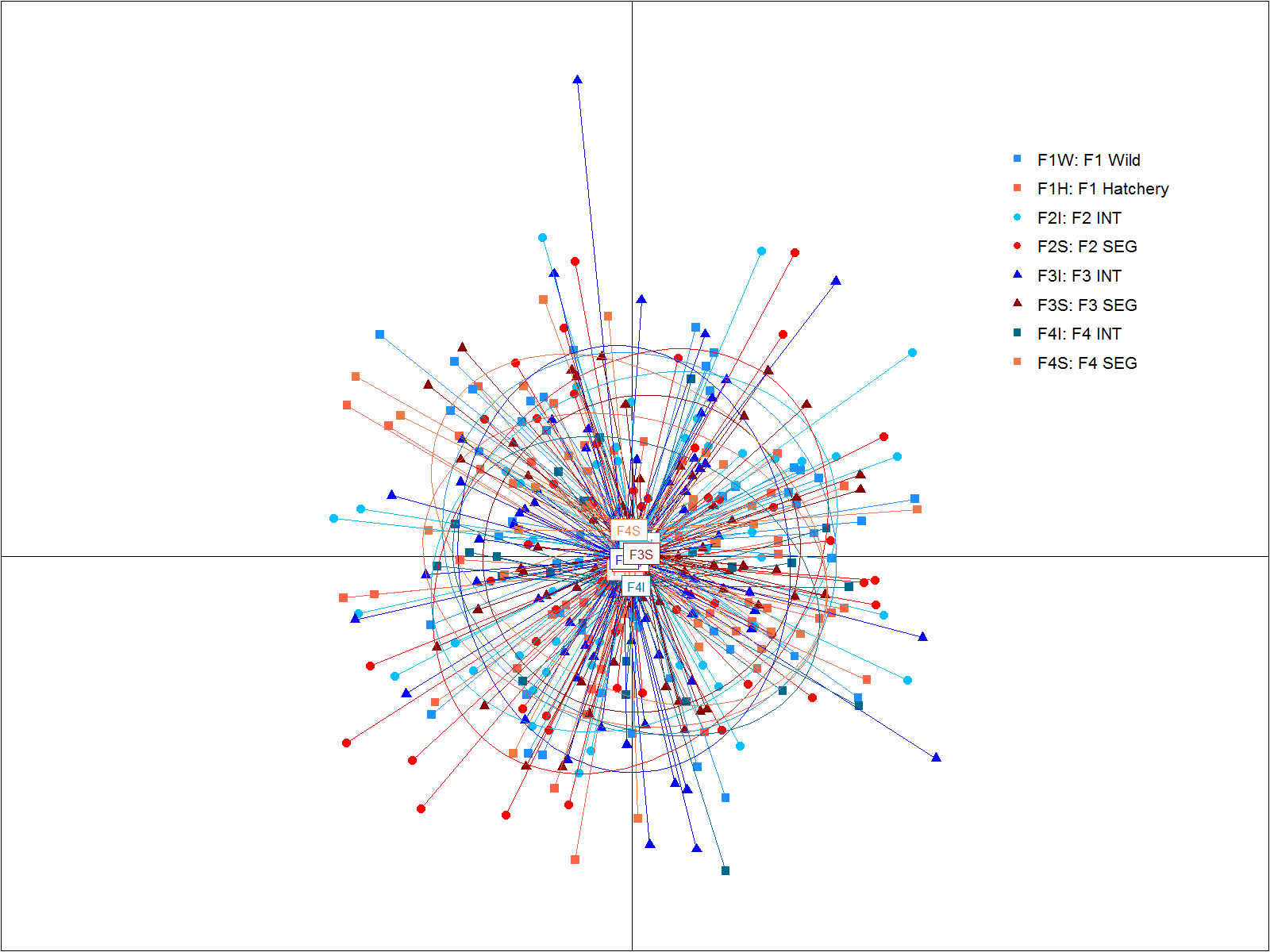


Figure S3. Plot of individuals along the first and second principal components (4.9% and 4.8% variation explained, respectively) from a principal components analysis conducted on genotypes at 68 loci predictive of spawn timing, after correcting for confounding factors. Points represent individuals, with lines connecting each individual to their respective population mean. Ellipses are shown to illustrate the dispersion of each population. The four generations of the integrated (INT) and segregated (SEG) hatchery lines are shown in shades of blue and red, respectively.


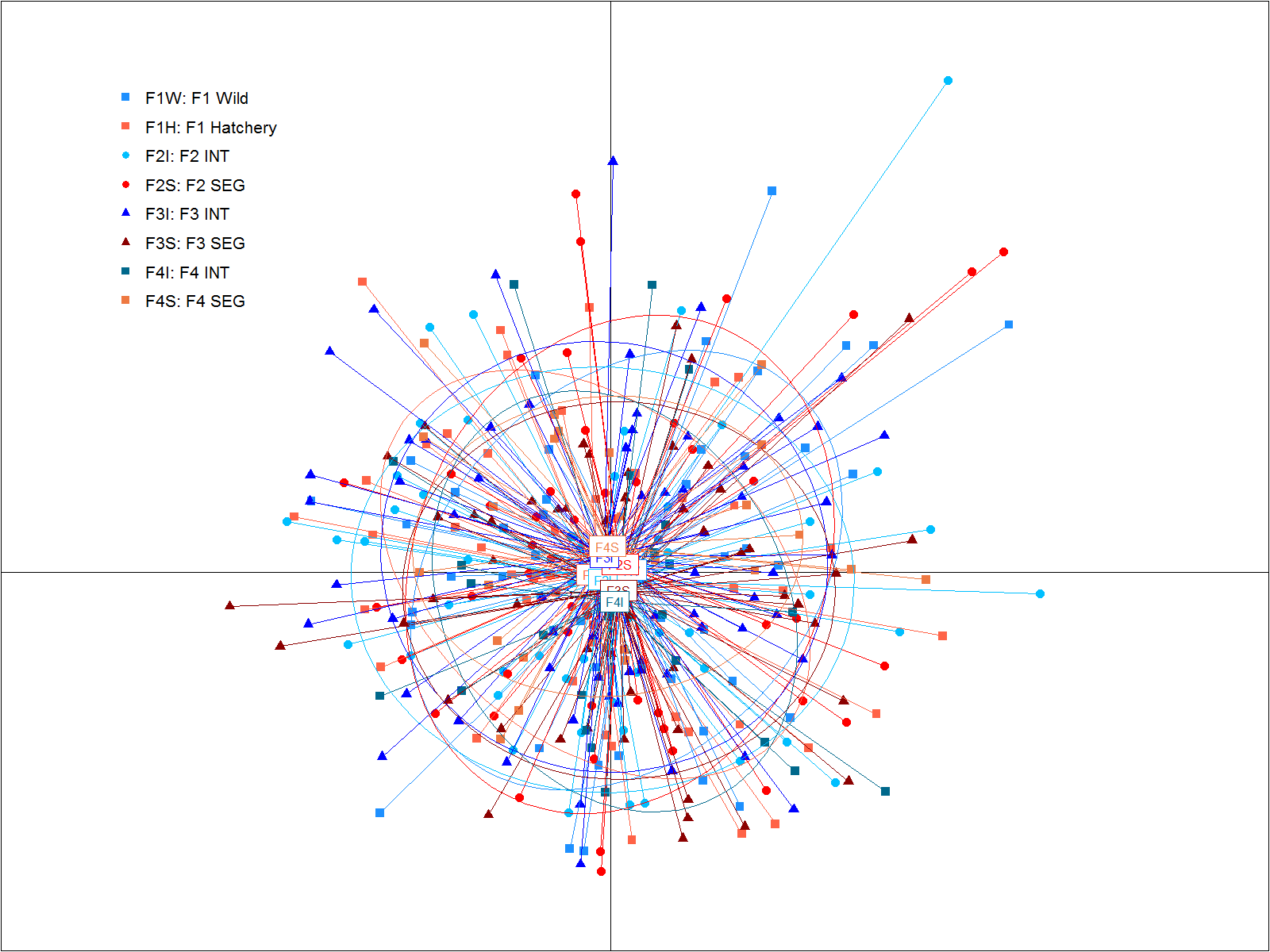


Figure S4. Plot of individuals along the first and second principal components (7.9% and 7.3% variation explained, respectively) from a principal components analysis conducted on genotypes at 35 loci predictive of daily growth coefficient (DGC), after correcting for confounding factors. Points represent individuals, with lines connecting each individual to their respective population mean. Ellipses are shown to illustrate the dispersion of each population. The four generations of the integrated (INT) and segregated (SEG) hatchery lines are shown in shades of blue and red, respectively.


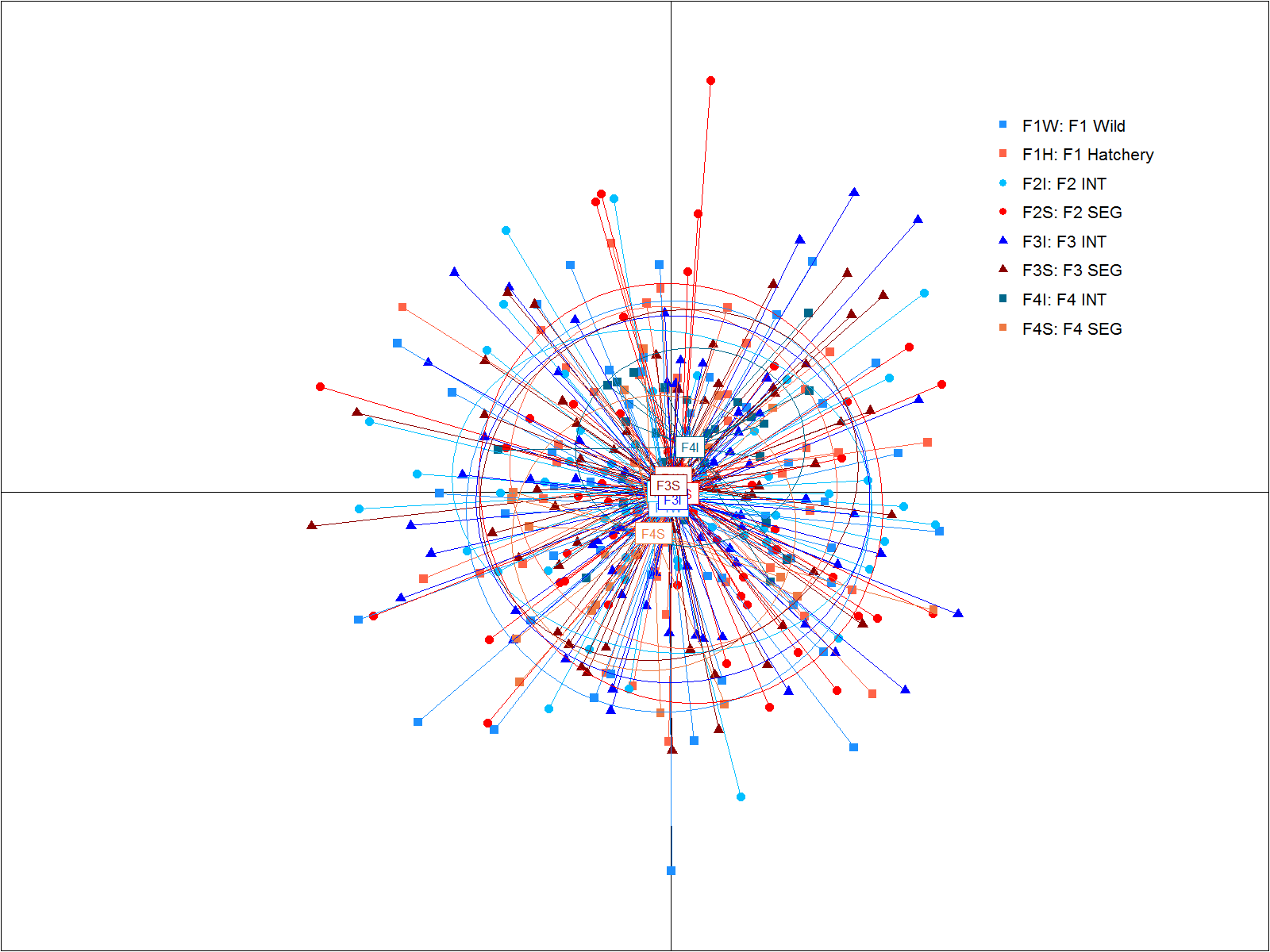


Figure S5. Plot of individuals along the first and second principal components (7.8% and 7.2% variation explained, respectively) from a principal components analysis conducted on genotypes at 37 loci predictive of weight at Roza Dam, after correcting for confounding factors. Points represent individuals, with lines connecting each individual to their respective population mean. Ellipses are shown to illustrate the dispersion of each population. The four generations of the integrated (INT) and segregated (SEG) hatchery lines are shown in shades of blue and red, respectively.


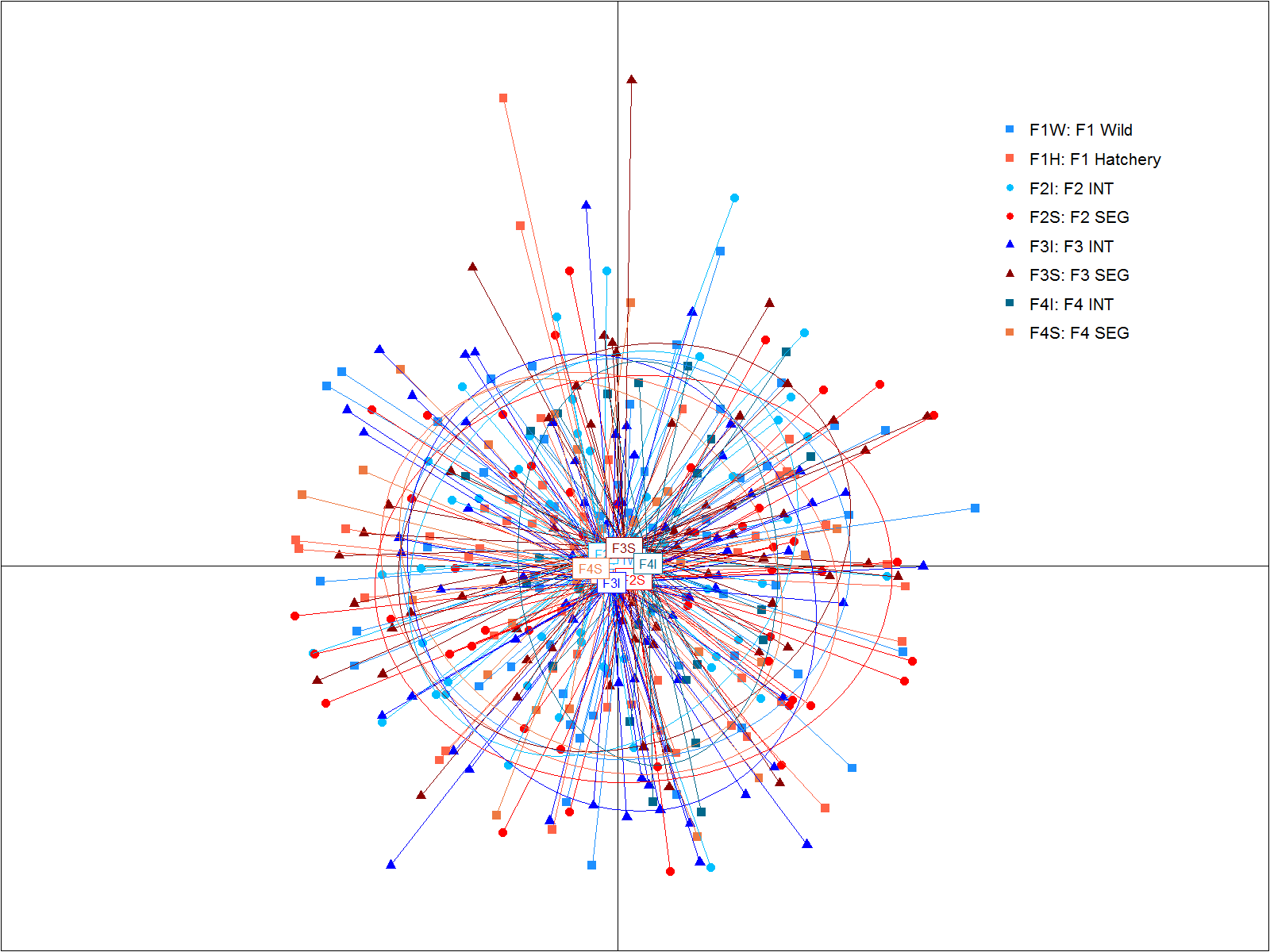


Figure S6. Plot of individuals along the first and second principal components (9.9% and 9.1% variation explained, respectively) from a principal components analysis conducted on genotypes at 26 loci predictive of return timing, after correcting for confounding factors. Points represent individuals, with lines connecting each individual to their respective population mean. Ellipses are shown to illustrate the dispersion of each population. The four generations of the integrated (INT) and segregated (SEG) hatchery lines are shown in shades of blue and red, respectively.


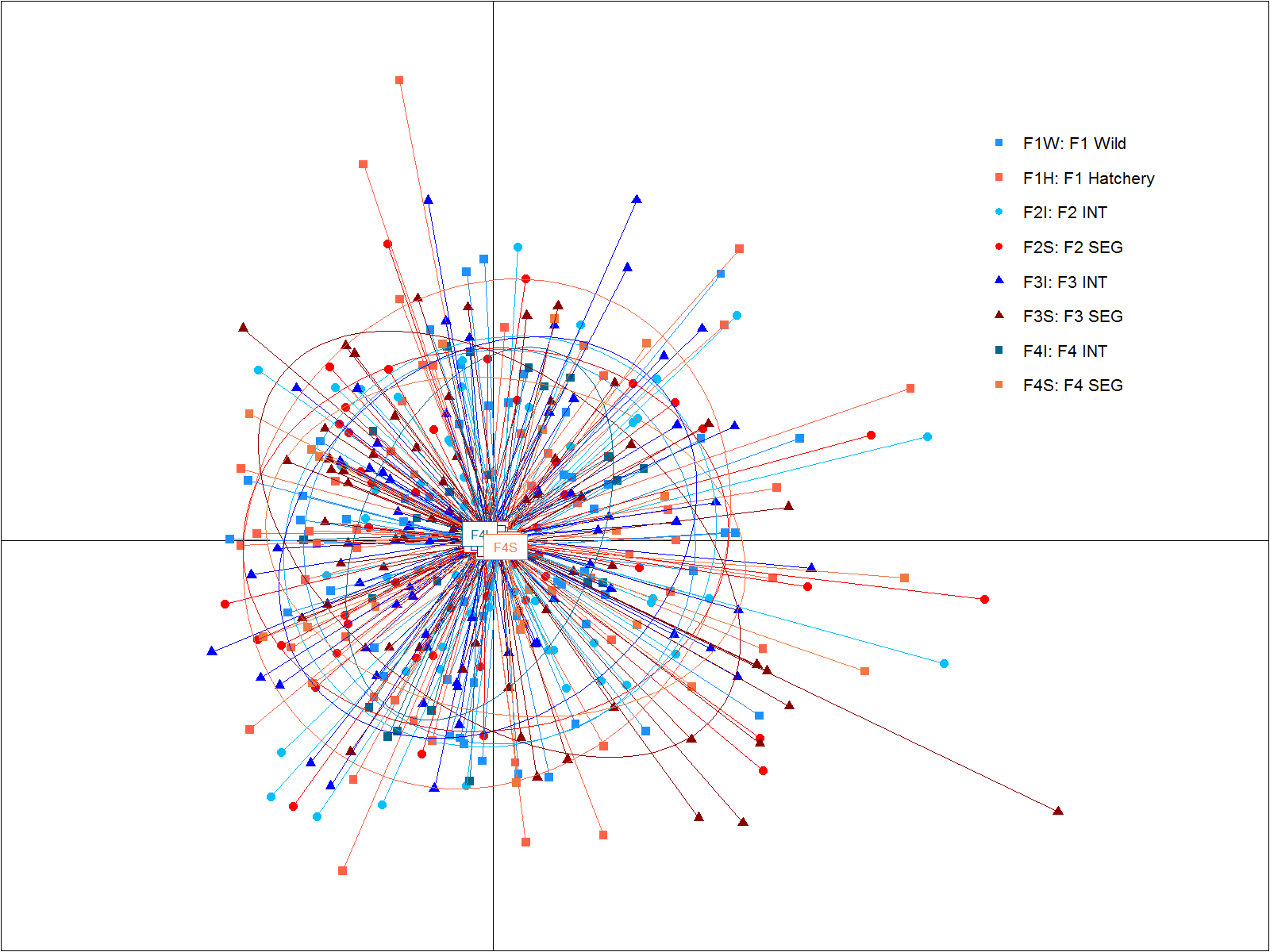


Figure S7. Plot of individuals along the first and second principal components (7.6% and 6.7% variation explained, respectively) from a principal components analysis conducted on genotypes at 30 loci predictive of age at maturity, after correcting for confounding factors. Points represent individuals, with lines connecting each individual to their respective population mean. Ellipses are shown to illustrate the dispersion of each population. The four generations of the integrated (INT) and segregated (SEG) hatchery lines are shown in shades of blue and red, respectively.


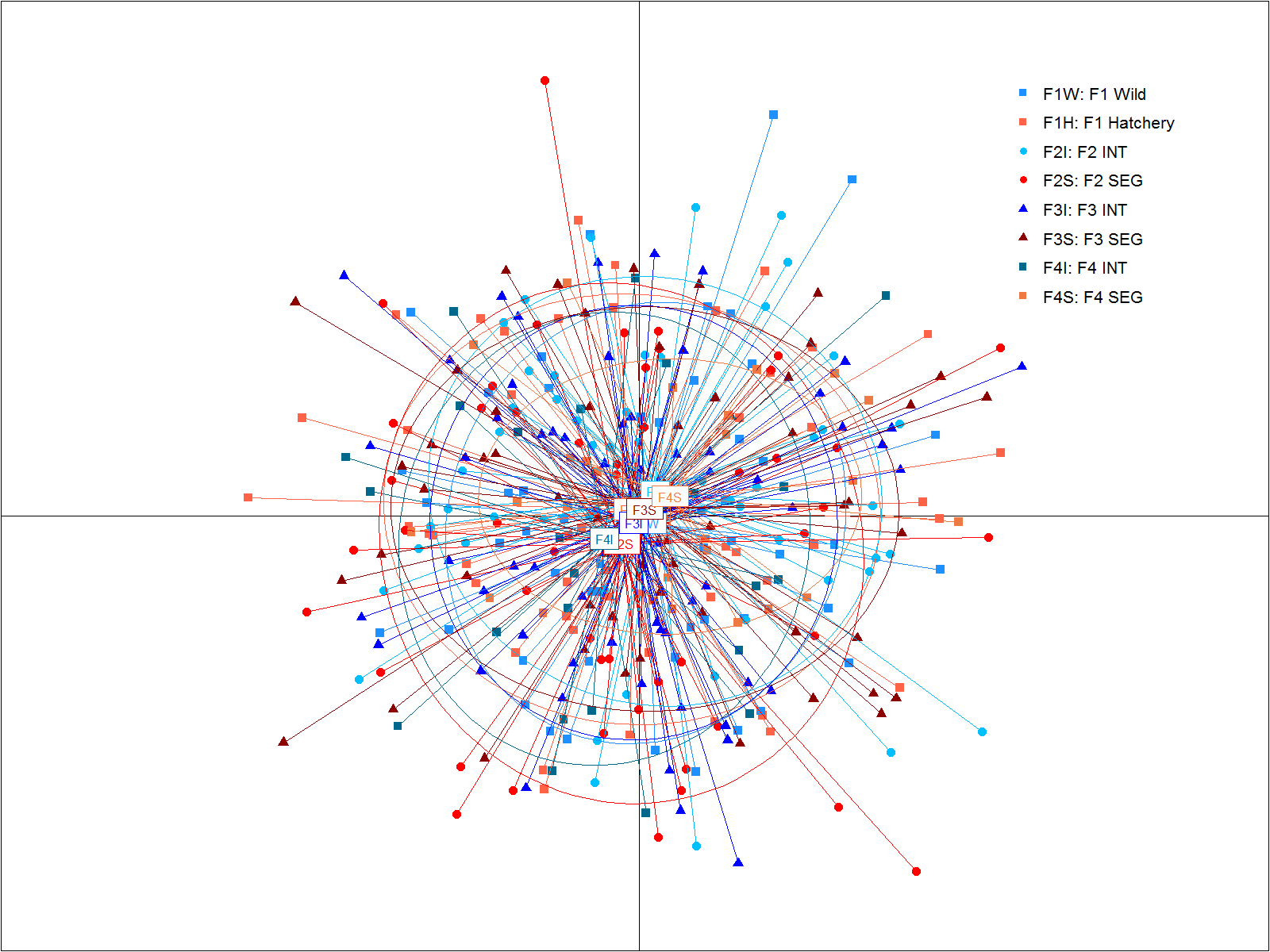


Figure S8. Plot of individuals along the first and second principal components (7.5% and 6.9% variation explained, respectively) from a principal components analysis conducted on genotypes at 44 loci predictive of fork length at Roza Dam, after correcting for confounding factors. Points represent individuals, with lines connecting each individual to their respective population mean. Ellipses are shown to illustrate the dispersion of each population. The four generations of the integrated (INT) and segregated (SEG) hatchery lines are shown in shades of blue and red, respectively.

Comparisons between trait-associated loci and previously identified outlier loci (Waters et al., 2015; Waters et al., 2017) revealed several regions where the groups overlapped or were in close proximity to each other.

*
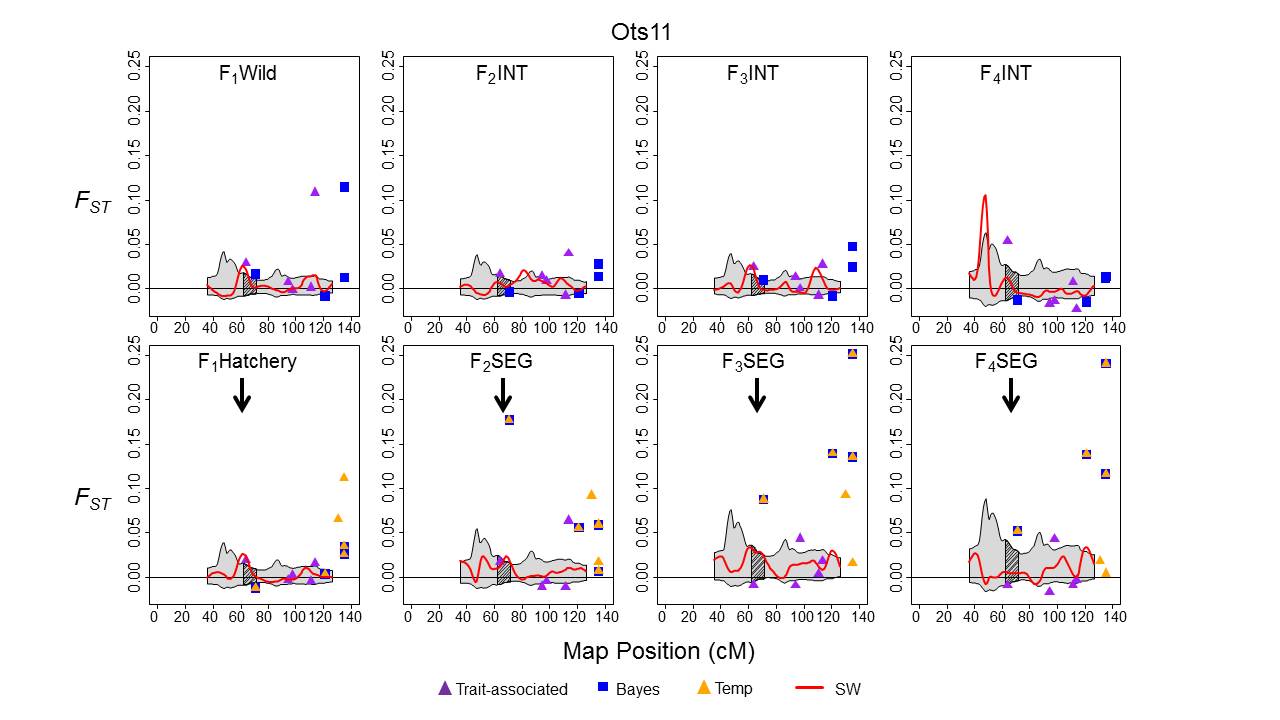
*

Figure S9. Overlap of trait-associated loci, outlier loci, and regions of chromosome Ots11 that show signatures of adaptive divergence based on measures of pairwise *F_ST_* between each generation of each Chinook salmon hatchery line and the P_1_ founders. The results are given for the integrated (top panel) and segregated (bottom panel) hatchery lines through the F_1_, F_2_, F_3_, and F_4_ generations. Blue squares are loci that were identified as outliers with *Bayescan*, orange triangles are outliers identified by *F_TEMP_*, a method designed to detect selection in a single population over time, and purple triangles are trait-associated loci. The red line represents the kernel smoothed moving average of *F_ST_* and the grey shaded area is the 95% confidence interval. The centromere of the chromosome is shaded with diagonal black lines. Genomic regions exhibiting significant levels of divergence (i.e. outlier regions) from the P_1_ founders occur where the moving average of *F_ST_* exceeds the 95% confidence intervals. Segments from 57 to 72 cM exhibited significantly elevated divergence in the F_1_, F_2_, and F_3_ generations of the segregated line and also contained a locus linked to age at maturity and an outlier locus. The black arrows mark this region in each generation. Part of the region also exhibited significant divergence in the F_1_ and F_3_ generations of the integrated line but did not contain any outlier loci.

*
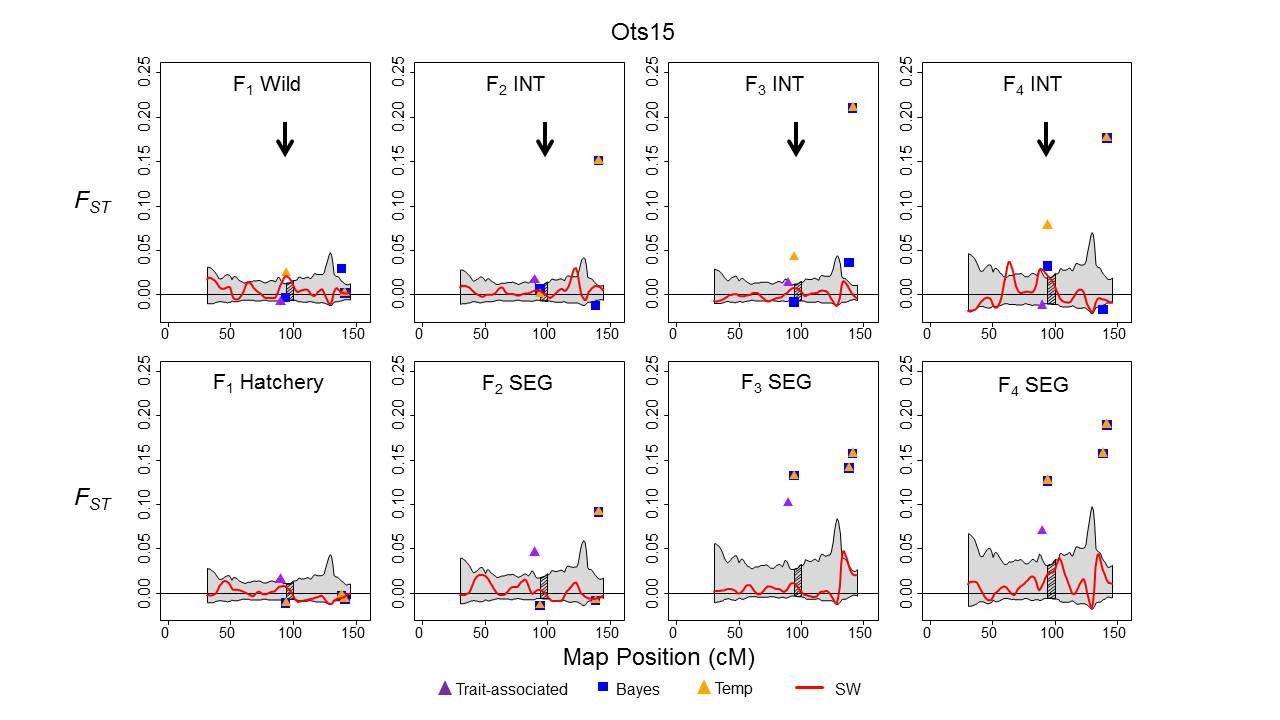
*

Figure S10. Overlap of trait-associated loci, outlier loci, and regions of chromosome Ots15 that show signatures of adaptive divergence based on measures of pairwise *F_ST_* between each generation of each Chinook salmon hatchery line and the P_1_ founders. The results are given for the integrated (top panel) and segregated (bottom panel) hatchery lines through the F_1_, F_2_, F_3_, and F_4_ generations. Blue squares are loci that were identified as outliers with *Bayescan*, orange triangles are outliers identified by *F_TEMP_*, a method designed to detect selection in a single population over time, and purple triangles are trait-associated loci. The red line represents the kernel smoothed moving average of *F_ST_* and the grey shaded area is the 95% confidence interval. The centromere of the chromosome is shaded with diagonal black lines. Genomic regions exhibiting significant levels of divergence (i.e. outlier regions) from the P_1_ founders occur where the moving average of *F_ST_* exceeds the 95% confidence intervals. Segments from 86 to 98 cM exhibited significantly elevated divergence in the F_1_ and F_4_ generations of the integrated line and also contained a locus linked to fork length and two outlier loci. The black arrows mark this region in each generation.

*
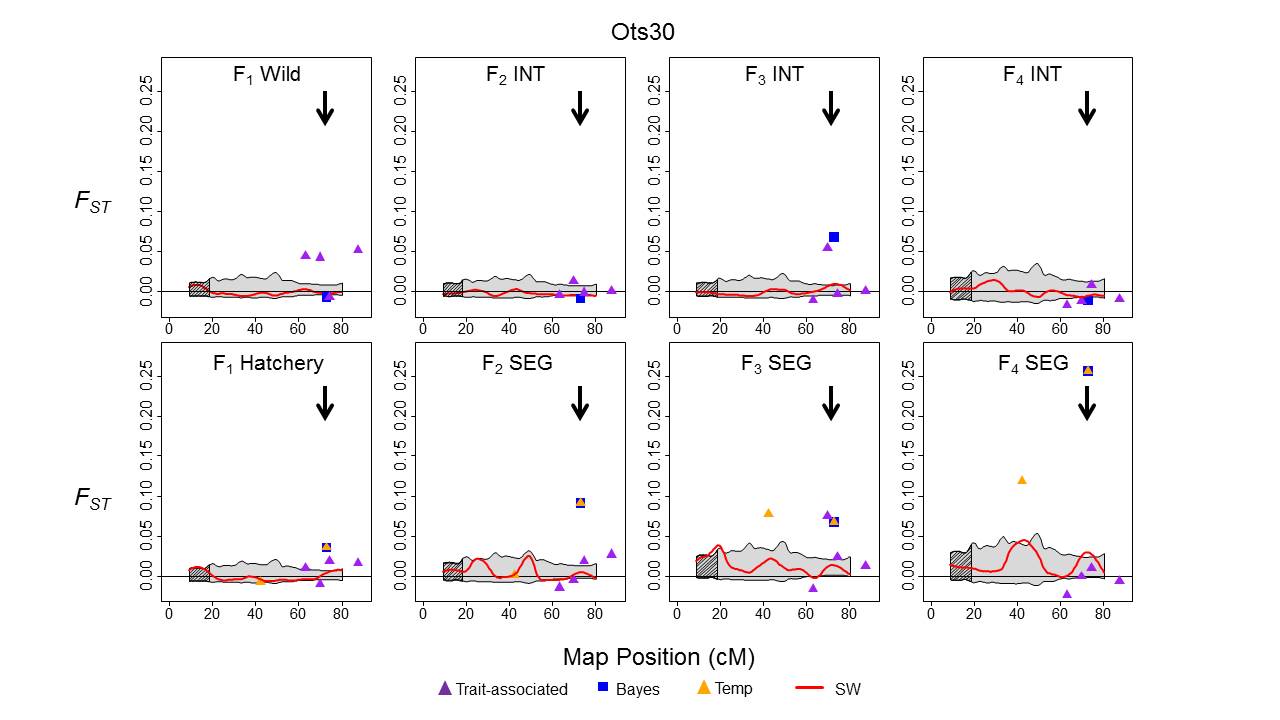
*

Figure S11. Overlap of trait-associated loci, outlier loci, and regions of chromosome Ots30 that show signatures of adaptive divergence based on measures of pairwise *F_ST_* between each generation of each Chinook salmon hatchery line and the P_1_ founders. The results are given for the integrated (top panel) and segregated (bottom panel) hatchery lines through the F_1_, F_2_, F_3_, and F_4_ generations. Blue squares are loci that were identified as outliers with *Bayescan*, orange triangles are outliers identified by *F_TEMP_*, a method designed to detect selection in a single population over time, and purple triangles are trait-associated loci. The red line represents the kernel smoothed moving average of *F_ST_* and the grey shaded area is the 95% confidence interval. The centromere of the chromosome is shaded with diagonal black lines. Genomic regions exhibiting significant levels of divergence (i.e. outlier regions) from the P_1_ founders occur where the moving average of *F_ST_* exceeds the 95% confidence intervals. The region from 69 to 76 cM exhibited significantly elevated divergence in the F_4_ generation of the segregated line and also contained a locus linked to age at maturity, a locus linked to fork length, and one outlier locus. Part of the region also exhibited significant divergence in the F_3_ generation of the integrated line; this segment contained the locus linked to fork length and one outlier locus. The black arrows mark this region in each generation.

**Literature cited**

Brieuc, M. S. O., Waters, C. D., Seeb, J. E., & Naish, K. A. (2014). A dense linkage map for Chinook salmon (*Oncorhynchus tshawytscha*) reveals variable chromosomal divergence after an ancestral whole genome duplication event. *G3-Genes Genomes Genetics, 4*, 447-460.

Scheet, P., & Stephens, M. (2006). A fast and flexible statistical model for large-scale population genotype data: Applications to inferring missing genotypes and haplotypic phase. *American Journal of Human Genetics, 78*, 629-644.

Waters, C. D., Hard, J. J., Brieuc, M. S. O., Fast, D. E., Warheit, K. I., Waples, R. S., . . . Naish, K. A. (2015). Effectiveness of managed gene flow in reducing genetic divergence associated with captive breeding. *Evolutionary Applications, 8*, 956-971.

Waters, C. D., Hard, J. J., Brieuc, M. S. O., Fast, D. E., Warheit, K. I., Waples, R. S., . . . Naish, K. A. (2017). What can genomics tell us about the success of enhancement programs in anadromous Chinook salmon? A comparative analysis across four generations. Supplementary Material for Bernatchez et al. (2017) Harnessing the power of genomics to secure the future of seafood. *Trends in Ecology & Evolution*, 665-680.
